# Telehealth-Delivered Mindfulness-Based Intervention: Protocol for a Randomized Clinical Trial for Individuals With Alcohol Use Disorder

**DOI:** 10.2196/92198

**Published:** 2026-05-01

**Authors:** Megan Kirouac, Daniel S Otero, David I K Moniz-Lewis, Sarah Bowen, Corey R Roos, Christine Vinci, Angel R Vasquez, Matison McCool, Frank J Schwebel, Roberta Chavez, Amber Martinez, Rena Quintana, Rachel Olson, Katie Witkiewitz

**Affiliations:** 1 Center on Alcohol, Substance use, And Addictions (CASAA), University of New Mexico, 2650 Yale Blvd SE, Albuquerque, NM, 87106, United States, 1 5055851686; 2 School of Graduate Psychology, Pacific University, Hillsboro, OR, United States; 3 Yale University School of Medicine, New Haven, CT, United States; 4 Department of Health Outcomes and Behavior, Moffitt Cancer Center, Tampa, FL, United States

**Keywords:** alcohol use disorder, recovery, behavioral health, mindfulness-based relapse prevention, mutual support groups, hybrid effectiveness-implementation trial, continuing care

## Abstract

**Background:**

Globally, approximately 8.6% of people will meet criteria for alcohol use disorder (AUD) in their lifetimes, with 2.2% meeting criteria for AUD in the past 12 months. In the United States, AUD prevalence is even greater, with 13.9% meeting criteria in the past 12 months. Effective treatments for AUD exist, although most people receive help through mutual support groups (ie, Alcoholics Anonymous [AA]). However, AA and other mutual support programs may not be desirable for all individuals, particularly those who do not seek abstinence-based approaches. Treatments that support reductions in drinking have been shown to be as effective as abstinence-based treatments in reducing alcohol-related harms, and may be more appealing to a broader range of people. Mindfulness-based interventions may be particularly effective in supporting long-term recovery, whole-person health, and functioning for those with abstinent and nonabstinent recovery goals.

**Objective:**

This study is designed to test a novel rolling group-based mindfulness-based relapse prevention (MBRP) delivered via videoconferencing, focused on drinking reduction goals and whole-person functioning.

**Methods:**

This study is a hybrid type 1 effectiveness-implementation design to prospectively test the effectiveness of MBRP and to identify barriers and facilitators of group participation to inform future implementation of MBRP as continuing care. Individuals with AUD (n=470) interested in stopping or reducing their drinking, meet criteria for AUD based on a symptom checklist, report heavy drinking at least once in the past 6 months, consent and understand study procedures in English, and provide a valid US mailing address are recruited nationwide via online sources in the United States. Participants are randomized to either MBRP groups via Zoom (Zoom Video Communications, Inc) or referral to online mutual support (eg, AA). All participants receive an individual orientation session delivered via Zoom that includes brief motivational interviewing and an overview of their assigned condition and next steps (MBRP condition: overview of the MBRP groups and instructions on participating in MBRP via Zoom; referral condition: referral to online mutual support groups). Participants provide blood samples for phosphatidylethanol testing at baseline and 3-year follow-up and complete self-report measures of psychosocial functioning, alcohol and other drug use, addiction cycle domains, and previously established predictors of recovery every 6 months for 3 years.

**Results:**

Recruitment began on September 13, 2023, and the last recruited participant was randomized on March 20, 2025. Follow-up data collection is ongoing with all 6-month follow-ups completed (86% retention).

**Conclusions:**

This study evaluates the effectiveness and mechanisms of MBRP delivered via videoconferencing, compared with referral to online mutual support groups, in supporting recovery among individuals with AUD. It also examines the reach, effectiveness, adoption, implementation, and maintenance of MBRP as an accessible, freely available continuing care option to support long-term recovery from AUD.

## Introduction

### Background

Globally, approximately 8.6% of people will meet criteria for alcohol use disorder (AUD) in their lifetimes, with 2.2% meeting criteria for AUD in the past 12 months [1]. In the United States, where the current trial was conducted, approximately 29% of US Americans will meet criteria for AUD in their lifetimes, with 13.9% meeting criteria for AUD in the past 12 months. The economic burden of excessive alcohol use is also staggering, costing the US economy at least US $249 billion annually [2]. Recent data from the Centers for Disease Control [3] indicated that 1 in 8 deaths among adults aged 20 to 64 years from 2015 to 2019 were attributable to excessive alcohol use, and this number increased to 1 in 5 deaths among adults younger than 49 years. In states with more rural and underserved populations, the rates of mortality were approximately 1 in 5 deaths among all adults aged 20 to 64 years. There are many drivers to high rates of morbidity and mortality due to alcohol, including the affordability and accessibility of alcohol, lack of access to treatment services and continuing care, social acceptability of alcohol use, and stigma associated with AUD and help-seeking [4-9].

Although effective pharmacological and psychosocial treatments for AUD exist [10], many individuals do not receive them and most are treated via mutual support group participation or community rehabilitation programs [11]. Approximately 80% of individuals with AUD never seek treatment, and not wanting to stop drinking is a common barrier to seeking treatment [9]. Many treatment programs in the United States are abstinence-based; yet, treatments that focus on reductions in drinking have been shown to be as effective in reducing alcohol-related harms [12]. Recent studies in population-based and clinical samples indicate significant health benefits from drinking reductions, without total abstinence [13-16]. Consistent with these data, the National Institute on Alcohol Abuse and Alcoholism (NIAAA) recently operationally defined recovery as remission from AUD, cessation of heavy drinking (defined as no more than 14 [males] or 10 [females] standard drinks per week and no more than 4 [males] or 3 [females] standard drinks on a single day), and improvements in functioning and well-being [17]. Following the *DSM-5* (*Diagnostic and Statistical Manual for Mental Disorders* [Fifth Edition]) definition, AUD in remission is classified as no longer meeting criteria for AUD (ie, 2 or more symptoms) [18]. Expanding the definition of recovery to include nonabstinent outcomes may increase provider awareness and acceptance of nonabstinent treatment goals; it may further increase treatment-seeking among those with AUD who are unwilling or unable to abstain but willing to reduce their drinking, and ultimately reduce the public health burden of untreated AUD [19,20].

AUD is often characterized as a “chronic brain disease,” and approximately 20%‐30% of individuals return to frequent, heavy drinking after treatment [10]. Recent translational work has proposed 3 stages of addiction, called the addiction cycle [21,22], that are based on behavioral and neuroadaptations observed in alcohol and other substance use disorders. According to the addiction cycle, in the binge or intoxication stage of AUD, drinking is primarily maintained through positive reinforcement and seeking euphoria. During the withdrawal or negative affect stage, alcohol is primarily consumed to relieve aversive negative affective states that may arise from repeated withdrawal. Over time, alterations in reward processing and executive control contribute to increasingly habitual alcohol seeking with diminished inhibitory regulation (preoccupation or anticipation stage). Parsing heterogeneity of AUD based on neuroadaptations is useful for translational work in modeling brain-behavior relationships and holds promise for delivering continuing care that is tailored to specific dysfunctions [10,23]. These brain-behavior relationships may partially explain a commonly observed phenomenon in individuals in early recovery, “quitting is not nearly as difficult as staying quit” [24]. Despite this, treatment is often delivered in discrete episodes, leaving a significant need for more continuing care options to support long-term recovery from AUD [25,26].

Alcoholics Anonymous (AA) and other 12-step programs have been shown to be highly effective in supporting abstinence [27], and are a tremendously valuable option for those interested in abstinence-based recovery. Decades of research have found AA to be effective for a range of populations [27-29] and to support recovery through a variety of mechanisms, including spiritual, cognitive, and social support mechanisms [30-35]. Additional benefits of AA and mutual support programs include accessibility and availability at little or no cost [27,36]. However, AA and other 12-step programs may not be optimal for all individuals, particularly those who do not seek abstinence or who do not experience a sense of belonging in such groups (often women and members of minoritized racial and ethnic groups) [37-41]. Further, mutual support groups appear to be predominantly effective via increases in social network support [42], which may be insufficient for those with more severe distress and emotional regulation impairments [43]. Altogether, the literature highlights a need for alternative, evidence-based continuing care options that (1) target many pathways to recovery from AUD and whole-person recovery, (2) are effective in targeting the neuroadaptations observed among those with AUD, and (3) are widely accessible and available at little or no cost to address the public health burden of alcohol use.

Mindfulness-based relapse prevention (MBRP) has demonstrated efficacy in reducing heavy drinking in several randomized clinical trials [44-47] and holds promise as a continuing care intervention that supports long-term whole-person recovery. MBRP was developed as an aftercare intervention to help prevent relapse to heavy drinking. The largest effect sizes have been shown when MBRP is delivered as continuing care [48]. Mindfulness-based interventions foster awareness of present-moment internal experiences, including thoughts, emotions, and sensations, as well as attention to the perception of elements in the surrounding environment (eg, alcohol cues and risky environments). MBRP explicitly targets the ability to stay present with distressing experiences without reactivity. For example, in the “urge surfing practice,” [49] clients are instructed to imagine or recall a situation in which they experience intense stress or craving and to bring mindful awareness to the sensations, experiences, thoughts, and emotions that arise, and to stay with these contents of awareness, rather than engaging in avoidance or escape behaviors (ie, not distracting oneself or attempting to make these contents “go away”). In this process, there is an opportunity to notice that these phenomena eventually dissipate without direct intervention. Another practice used throughout the program is the Stop-Observe-Breathe-Expand-Respond (SOBER) space [49], a brief breath mindfulness practice that can be used in moments of distress or intense craving. The heightened awareness facilitated by mindfulness training can be contrasted with “automatic” cognitive and behavioral reactions (ie, “autopilot”) that often occur without conscious awareness and increase the risk of relapse [50]. Further, MBRP is focused on improving broader domains of whole-person recovery and life functioning. For example, in a prior study of MBRP [45], we found that none of those who received MBRP as part of programming in a residential treatment program were discharged to living on the street or in their car, an outcome for 17% of those in the treatment-as-usual condition. Further, this study found that individuals in MBRP had fewer legal and social problems than those in the treatment-as-usual condition [45]. Other studies have highlighted the role MBRP may play in addressing other whole-person recovery facets, including anxiety and depression [51], stress [52], and quality of life [53].

MBRP also holds promise for targeting the specific dysfunctions of the addiction cycle. In addition to targeting whole-person functioning, MBRP explicitly targets 3 key aspects of addiction that map onto constructs of the neurobiologically-informed addiction cycle: craving (incentive salience), negative affect (negative emotionality), and inhibitory control over behavioral responses (executive function). Specifically, MBRP has been shown to improve cognitive control [54], reduce craving [55,56], and reduce reactivity to negative emotions [57]. It is hypothesized that MBRP may support longer-term recovery from AUD via a broader focus on whole-person functioning and by targeting these addiction cycle domains.

Similar to mutual support programs, MBRP can be delivered remotely. We previously tested MBRP via Zoom (Zoom Video Communications, Inc) videoconferencing in a pilot trial [58,59], and are currently conducting 2 large multisite randomized clinical trials of MBRP via videoconferencing (Zoom and VA Video Connect [US Department of Veterans Affairs]) for people with chronic pain and opioid use disorder. The delivery of MBRP via Zoom provides a unique opportunity to offer MBRP as a nationwide continuing care program to support long-term recovery from AUD among individuals who have made a drinking change attempt. Importantly, MBRP can be delivered at little to no cost. Consistent with core and enduring traditions of Buddhism—from which mindfulness practice (and thus MBRP) derives—these teachings are traditionally offered freely. Importantly, MBRP has been developed in and is supported by this tradition such that developers and facilitators of MBRP groups often provide services at no cost. The senior author and her team have been delivering MBRP groups throughout the Albuquerque metro area, and more recently nationwide via Zoom, at no cost to group attendees since 2014.

### Objectives

The goal of this study is to examine the effectiveness of MBRP delivered via Health Insurance Portability and Accountability Act (HIPAA)–compliant Zoom in promoting whole-person recovery from AUD, and to examine whether MBRP in this context affects mechanisms of behavior change based on the neurobiologically-informed addiction cycle domains as predictors of AUD recovery. Further, this study uses a hybrid effectiveness-implementation type 1 design [60] to prospectively test the effectiveness of MBRP, as well as identify barriers and facilitators of MBRP group participation and facilitation. Individuals (n=470) who have recently expressed interest in changing their drinking or have recently stopped or reduced drinking with or without formal treatment are randomized to receive either MBRP or referral to online mutual support using urn randomization to balance groups on patient birth sex and severity of AUD. We hypothesize that receiving MBRP, versus online mutual support, will be associated with greater odds of recovery from AUD up to 3 years following a drinking change attempt, as indicated by AUD remission, cessation of heavy drinking, and improvements in functioning and well-being. We also test whether addiction cycle domains mediate the effect of treatment on recovery, and hypothesize that MBRP will be associated with greater improvements in executive functioning and self-efficacy, and reductions in incentive salience and negative emotionality in the 3 years following a change attempt, and these constructs predict recovery outcomes. Finally, using a hybrid type 1 effectiveness-implementation design, we examine facilitators and barriers to participation and engagement in MBRP, and assess the adoption, implementation, reach, effectiveness, and maintenance of MBRP via video telehealth as continuing care in communities nationwide. This hybrid effectiveness-implementation trial is referred to herein as the Telehealth Resources for Individualized Recovery (THRIVE) goals study.

## Methods

### Trial Design

The trial is a parallel group randomized clinical trial with individuals randomized to receive either MBRP or referral to online mutual support using urn randomization to balance groups on patient sex and severity of AUD. We propose a superiority design, whereby MBRP will be superior to referral to online mutual support.

### Study Setting

The study is conducted entirely online via Zoom videoconferencing software with participants recruited nationwide from communities in the United States. Using a hybrid effectiveness-implementation design, this study recruits 2 types of participants: “client participants” and “facilitator participants.” “Client participants” are participants who join the study with the goal of reducing or ceasing their alcohol use, contrasted with “facilitator participants” who participate in the study and facilitate the MBRP groups. Recruitment of “client participants” in the effectiveness arm of the study is conducted via online ads developed and administered by BuildClinical, as well as via the posting of recruitment flyers throughout communities in targeted locations (treatment agencies and oft-visited locations near treatment agencies in Albuquerque, New Mexico), online and social media recruitment sites, and links for recruitment on the Center on Alcohol, Substance Use, and Addictions (CASAA) homepage. MBRP facilitators are recruited via an announcement to a listserv of individuals who had previously been trained in MBRP, as well as those individuals who were already working with the study principal investigator (PI) in facilitating MBRP groups. A subset of these MBRP facilitators is recruited for the implementation arm of the study and is herein referred to as “facilitator participants.”

### Eligibility Criteria

Textbox 1 presents the criteria necessary for client participants to be eligible to participate in the effectiveness arm of this study.

Textbox 1. **Eligibility criteria for the efficacy arm.**

**Inclusion criteria**

- At least 18 years old
- Received alcohol use disorder (AUD) treatment or made a self-change attempt to reduce or stop drinking in the past 3 months, or is interested in reducing or stopping drinking
- Meet criteria for a current AUD, as defined by a score of 2 or greater on the Alcohol Symptom Checklist [60]
- Engaged in heavy drinking (more than 14 standard drinks per week or more than 4 drinks on a single day for men and more than 7 drinks per week or more than 3 drinks on a single day for women) in the past 6 months
- Able to understand all study procedures and able to consent in English
- Willing to use a personal smartphone or tablet that is connected to the internet, or being willing to use a study-provided tablet
- Have access to a valid US mailing address for receiving the dried blood spot card and reside in the United States

**Exclusion criteria**

- Have current symptoms of psychosis or mania
- Have a substance use disorder requiring a higher level of care than outpatient treatment (eg, severe AUD requiring inpatient detoxification)

The MBRP facilitators are recruited globally. To be eligible to participate in this study, facilitator participants must meet all the criteria displayed in Textbox 2.

Textbox 2. **Facilitator participant eligibility criteria for the implementation arm.**

**Inclusion criteria**

- Providing mindfulness-based relapse prevention (MBRP) groups as part of this research study. Note that not all MBRP facilitators are required to participate in the research, which is optional for MBRP facilitators.
- Able to understand all study procedures and able to consent in English.
- Providing MBRP groups as part of this research study. Note that not all MBRP facilitators are required to participate in the research, which is optional for MBRP facilitators.
- Able to understand all study procedures and able to consent in English.

### Ethical Considerations

#### Institutional Review

Study procedures have been reviewed and approved by the University of New Mexico (UNM) Institutional Review Board (IRB; Protocol no. 2212028742). Following IRB-approved procedures, participants were compensated via e-gift cards for the data they provided in study surveys and interviews. Study personnel are trained in research ethics following the Helsinki Ethical Principles for human subjects research. All study personnel uphold the ethical principles of beneficence and nonmaleficence, fidelity and responsibility, integrity, justice, and respect for people’s rights and dignity.

#### Confidentiality

Participant confidentiality is strictly held in trust by the investigators, study staff, and the study sponsor and their agents. This confidentiality is extended to cover testing of biological samples in addition to any study information relating to participants. The study protocol, documentation, data, and all other information generated are held in strict confidence. Data include information collected at baseline and follow-up visits, as well as treatment attendance data. The data include self-report measures. Study enrollment records are reviewed at least monthly. All data are linked by a study ID (ie, “Record ID”) number that is assigned by the study staff and used in REDCap (Research Electronic Data Capture) system to link records across visits. Limited identifying information is collected in order to ensure that data sequencing is accurate. Following data collection, the date of assessment data is recoded into the number of days after randomization. Thus, at the stage of data analysis, the data do not include identifying information. Further, all electronic databases will have other identifying information removed, and participants will only be identified by study ID number. No information concerning the study or the data is released to any unauthorized third party without prior written approval of the study sponsor. Authorized representatives of the National Institutes of Health (NIH) and the IRB may inspect all study documents and records required to be maintained by the investigator. To further protect the privacy of study participants, the Secretary of the Department of Health and Human Services (HHS) has issued a Certificate of Confidentiality (CoC) to all researchers engaged in biomedical, behavioral, clinical, or other human subjects research funded wholly or in part by the federal government. Recipients of NIH funding for human subjects research are required to protect identifiable research information from forced disclosure per the terms of the NIH Policy.

#### Informed Consent Procedures

Informed consent is initiated prior to the individual agreeing to participate in the study and continues throughout study participation. An extensive discussion of risks and possible benefits of study participation is provided to participants. An online virtual consent form detailing the study procedures and risks is given to the client participants at the time of appointment. For client participants to schedule a consent session, participants are asked not to use any alcohol or other intoxicating substances prior to or during their consent appointment. Client participants are advised that if study staff have reason to believe they may be intoxicated or otherwise impaired during the consent, assessment, or therapy session appointment, the appointment may be ended, or the client may be asked to leave and be rescheduled for a later date to ensure the safety and well-being of all study staff and participants in the study. Client participants are asked to show valid photo identification at the time of their consent appointment and pass a consent comprehension quiz for data quality controls. Client participants who participate are sent an IRB-approved informed consent form via REDCap. The prospective client participant is required to read and review the document or have the document read to them. The study staff describe the research study to the participant and answer any questions. Virtual visits require participant signature of the informed consent digitally via REDCap prior to completing any study-related activities. Participants are given the opportunity to discuss the study with research staff or think about it prior to agreeing to participate. A signed copy of the informed consent document is emailed to participants for their records. The consent process is documented in the research record via REDCap, including the following information: participant’s first and last name, if the participant consents to participate in the research study, if the participant consents to being audio recorded if placed in the MBRP group, and a signature field. Once the survey is submitted, it is stored with the study record with additional information regarding what consent version was used at the time of consent, and the time and date of the consent verification; a copy of the consent record can also be downloaded by the participant or requested to be shared with them at any time from the study staff. The final generated consent report shows a time variance between the date and time of the consent form and when it was added to the participant’s record.

We requested a waiver of documentation of consent for the facilitator surveys and individual interviews. Facilitator participants are presented with an informed consent webpage that highlights the purpose of the overall study, as well as information on the specific survey or interview, the study duration, confidentiality of their responses, their rights to skip or not respond to items if they are uncomfortable, and contact information of the research team and UNM IRB office. Minimal identifying information is collected for the surveys and interviews for the sole purpose of linking participant responses across surveys and interview data. All participants are assigned a unique identification code, and the master linking file, linking relevant identifying information (ie, name, phone, and email) with identification codes is stored separately from the deidentified data. These surveys and interviews represent no more than minimal risk to the participants. The identifiable information is discarded prior to transferring any raw data to usable data, and all data disseminated for the purpose of program evaluation within or outside the institution or to the scientific community is presented in aggregate form.

Both client and facilitator participants are informed that all data are stored indefinitely by the NIH or a data center selected by the NIH to enable future research use, but that name and other identifying details will not be kept with the final research data. Client participants are informed that the dried blood card sample will not be stored and only the phosphatidylethanol results from the dried blood spot sample are kept with research data. Client participants also consent that if they withdraw from this research study before it is finished, researchers may keep and continue to use data and samples that have already been collected.

### Interventions

#### Overview

MBRP has been tested in randomized clinical trials as a “stand-alone” treatment (without prior treatment) [61], as a primary treatment following detoxification and stabilization [45], as a “rolling group” component of residential treatment [62], and as an “aftercare” intervention following community treatment as usual [44,46]. This study is the first randomized trial of MBRP compared to referral to mutual support in the community and the first trial of MBRP as a continuing care intervention whereby individuals can attend as many sessions as they want (ie, “rolling group”). MBRP is especially well-suited for adaptation from a discrete treatment episode to continuing care, given its emphasis on establishing and maintaining a mindfulness practice that can flexibly be applied in any situation. This contrasts with other evidence-based treatments that emphasize teaching clients to use learned skills for specific high-risk situations (eg, cognitive behavioral therapy–based relapse prevention plans).

Given the focus on MBRP as a continuing care intervention, it was important to select a comparator that reflected existing continuing care models. Continuing care for AUD is largely based on referral to mutual support groups; therefore, referral to mutual support groups was selected as the comparator [63]. We chose a 2-group design and did not randomize to different kinds of mutual support (eg, SMART Recovery vs AA), such that client participants received referral to multiple mutual support options and were informed that they were welcome to select and try out any that interested them (eg, SMART Recovery and AA). Thus, this study assesses the effectiveness of MBRP as continuing care as compared with the typical approach of referring individuals to mutual help, regardless of which mutual help groups participants attend.

Interventions are not modified or discontinued by the research team for any reason, although trial participants could opt to discontinue attending the intervention conditions at any time. No concomitant care or additional interventions are prohibited during the trial.

Mutual support groups are freely delivered in the community and will continue throughout the trial. MBRP groups are offered throughout the duration of the study, as long as there is interest by at least 2 or more group members. If groups are not continued due to low census, and any one individual is interested in continuing to practice mindfulness, the research team will refer MBRP client participants to local (if available) or online Recovery Dharma groups, which will offer the opportunity for ongoing mindfulness practice related to recovery.

#### Orientation Session

For both intervention conditions, the first session is an individual orientation delivered via Zoom by a study team member. Orientation session leaders include the research coordinator, study coinvestigators, a postdoctoral fellow, and clinical psychology graduate students. This orientation session is approximately 30 minutes and includes brief motivational interviewing [64], an overview of the project timeline, an introduction to the assigned intervention condition, and instructions for collecting blood samples via a mailed dried blood spot card.

#### MBRP Condition

In addition to the elements described above, the orientation session for the MBRP condition includes a review of the Zoom environment, an opportunity to learn Zoom for group sessions (eg, applying virtual backgrounds, breakout rooms, video and mute functions, swapping speaker, and panel view); an overview of MBRP; and an introduction to a free, MBRP mobile app created specifically for this study.

MBRP is delivered by trained facilitators and uses an existing rolling group treatment manual that has demonstrated effectiveness [62] and has been used feasibly via Zoom since March 2020. Participant worksheets and home practices were adapted from the closed-format MBRP manual [49] and guided meditations were drawn from the PracticeMBRP and MindfulRP websites. The rolling group manual consists of 8 sessions of 60 minutes each, covering the content described in Table 1.

Each session begins with a brief mindfulness practice and a discussion of mindfulness concepts (eg, “what is mindfulness?”) and the role mindfulness may play in recovery. The discussion encourages participants to apply the practices learned in the groups to their ongoing recovery. Consistent with a continuing care model, participants can attend as many MBRP sessions as they want to, and for as long as they want. Although the session content repeats every 8 weeks, we have found in our implementation of the program in community settings that individuals continue to attend beyond the full 8 sessions. Although themes are repeated, participants bring new experiences to the session, decreasing the risk of mere repetition. The group composition also includes people in various stages of recovery and familiarity with the material, which can make for richer discussions among group members themselves.

MBRP groups are facilitated by experienced community clinicians and clinical psychology graduate students. Facilitators were trained by SB and KW, who have trained MBRP facilitators for the past 2 decades. All MBRP facilitators are required to have a personal mindfulness practice and experience with mental health or addiction counseling. Many groups are cofacilitated with 2 or more facilitators and all facilitators receive group supervision from SB. The MBRP sessions are audio-recorded and uploaded to REDCap. Fidelity ratings of all group sessions are completed by a trained research coordinator and 10% of sessions are randomly selected for double coding by a trained graduate student or research assistant. Treatment fidelity is assessed using an adapted version of the Mindfulness-Based Relapse Prevention Adherence and Competence Scale (MBRP-AC) [65]. MBRP facilitators (n=15) are also invited to participate in the qualitative evaluation of the program.

To support group engagement and attendance, MBRP group attendees are provided weekly and monthly reminders of MBRP group times and any updates (eg, cancellations due to holidays). Groups are flexible, offered based on patient availability and interest such that new group times are formed to better accommodate participant schedules.

**Table 1.** MBRP^a^ session content.

Session	Content
1	Eating meditation with a small piece of food (eg, dried fruit), introduction to the SOBER^b^ breathing space, and using mindfulness in daily life
2	Mindfulness of thinking and thoughts, breath meditation
3	Mindful movement, SOBER breathing space, mindfulness, and valued living
4	Body scan, developing a daily practice and common challenges therein, false refuge
5	Kindness practice
6	SOBER breathing space in a challenging situation
7	Mindfulness and acceptance of difficult emotions, the Guest House poem meditation, and reading
8	Urge surfing

a MBRP: mindfulness-based relapse prevention.

b SOBER: Stop-Observe-Breathe-Expand-Respond.

#### Referral to Online Mutual Support Condition

The orientation session for the referral group is the only intervention provided to the comparator group, consisting of an orientation to online mutual support programs: InTheRooms, AA online, and SMART Recovery. Referral sources for individuals who are in crisis (eg, calling 988) are offered, as well as information about optional mobile apps for those wanting mobile app support (eg, apps to track drinking).

Mutual support groups are free, peer-led organizations designed to help individuals with substance use disorders and other addiction-related problems. Mutual support groups often focus on communication and exchange of addiction and recovery experience and skills. Through shared activities and discussions, participants provide mutual support and learning among individuals facing similar challenges.

### Outcomes

#### Overview

All outcomes are assessed at each time point (baseline and at follow-up months: 6, 12, 18, 24, 30, and 36) and are studied over time with change from baseline to the last follow-up period as the analysis metric.

#### Primary Outcome

Given the focus of the interventions on continuing care for AUD, recovery from AUD was chosen as the primary outcome and is based on the most recent empirical definition of recovery developed by NIAAA [17]. This empirical definition of recovery is a binary (yes or no) outcome defined by achieving all three of the following: (1) remission from *DSM-5* AUD based on an 11-item AUD symptom checklist [66] (endorsing <2 criteria is AUD remission, excepting the craving criterion which may persist even during total abstinence); (2) cessation of heavy drinking (defined as not engaging in heavy drinking over the past 28 days with heavy drinking defined as 4 or more standard drinks per occasion for females, and 5 or more standard drinks per occasion for males, measured by the Timeline Followback [67]); and (3) improvements in functioning and well-being as measured by higher score (from baseline) on the World Health Organization (WHO) Quality of Life [68] domain scores (each of 26 items scored from 1 to 5 on a response scale, which are then transformed linearly to a 0‐100 scale) and the 36-item Short Form Health Survey (SF-36) [69] Mental Health Component Score (scored on a 0‐100 scale). Recovery is defined by the achievement of remission, cessation of heavy drinking, and improvements in functioning and well-being. The assessment of recovery is completed at all time points, with proportion change from baseline to the final follow-up period (3 years postbaseline) assessed as the primary outcome.

#### Secondary Outcomes: Mechanisms of Behavior Change

##### Overview

Several secondary outcomes are evaluated with the following measures completed at all time points (baseline 6, 12, 18, 24, 30, and 36 months), and the average change from baseline to the final follow-up period was assessed as the secondary outcome.

##### Reductions in WHO Risk Drinking Levels

The Timeline Followback [67] calendar method of assessing standard alcohol drinks consumed each day over the past month is used to calculate the WHO risk levels [70,71] based on sex-specific grams of alcohol consumed per day in the month prior to the assessment, with levels defined as low risk (0‐20 females or 0‐40 males), moderate risk (21‐40 females and 41‐60 males), high risk (41‐60 females and 61‐100 males), and very high risk (61+ female or 100+ males). We examine those who achieve at least a 1-level or at least a 2-level reduction in risk drinking levels. The reference group for the 1-level reduction is no change or increase in WHO risk drinking level and the reference group for the 2-level reduction is 1-level reduction, no change or increase in WHO risk drinking level.

##### Reductions in Other Drinking Outcomes

The Timeline Followback [67] calendar method of assessing standard alcohol drinks consumed each day over the past month is used to calculate reductions in several other drinking-related metrics. The Timeline Followback is administered by trained study staff via video call, entered into a visual calendar viewable by the participant, and then entered by trained staff into REDCap. Percent heavy drinking days is calculated as the number of heavy drinking days in the past month divided by the total number of days in that time period (typically 28 days, unless some days are missing), where heavy drinking is defined as 4 or more standard drinks for females and 5 or more standard drinks for males, and heavy drinking occasions are used to identify heavy drinking days. Drinks per drinking day is calculated using the total number of standard drinks consumed on each drinking day, and the number of standard drinks per drinking day is calculated as the total number of standard drinks consumed in the past month divided by the total number of days in that time period when drinking occurred. Percent drinking days is calculated as the number of drinking days in the past 90 days divided by the total number of days in that time period, with drinking occasions used to identify drinking days.

##### Patient-Reported Outcomes Measurement Information System Measures

Several secondary outcomes are included from the Patient-Reported Outcomes Measurement Information System (PROMIS) [72-74], including alcohol negative consequences [75], Patient-Reported Outcomes Measurement Information System Preference (PROPr) score [76,77], and the PROMIS Meaning and Purpose measure [78]. The alcohol negative consequences measure includes 7 items scored from 1 (never) to 5 (almost always) that assess negative consequences from alcohol use (eg, I used poor judgment when I drank). Higher scores indicate more negative consequences. The PROPr combines scores from 7 PROMIS domains (cognitive function, depression, fatigue, pain interference, physical function, ability to participate in social roles and activities, and sleep disturbance) into a single health utility score. Higher scores indicate better health.

PROPr combines scores from 7 PROMIS domains (cognitive function, depression, fatigue, pain interference, physical function, ability to participate in social roles and activities, and sleep disturbance) into a single health utility score. Higher scores indicate better health. The Meaning and Purpose measure includes 8 items scored from 1 (not at all) to 5 (very much) that assess one’s sense that life has purpose and there are good reasons for living (eg, my life has meaning). Higher scores indicate greater purpose in life.

##### Penn Alcohol Craving Scale

The Penn Alcohol Craving Scale [79] is a 5-item measure of overall craving for alcohol. Responses range from 0 to 6, where 0 represents the absence of the craving and 6 represents the maximum intensity or frequency of the craving. Higher scores on the Penn Alcohol Craving Scale reflect more severe alcohol craving.

##### AUD Symptoms

The AUD Symptom Checklist [66] is a binary (yes or no), 11-item checklist of the symptoms of AUD experienced in the past 12 months (baseline assessment) and past 6 months (every 6 months at follow-up assessments). Higher scores indicate more symptoms of AUD.

##### Quality of Life

The World Health Organization Quality of Life–Brief Version (WHOQOL-BREF) [68] measure is a 26-item measure consisting of 4 domains: physical health (7 items), psychological health (6 items), social relationships (3 items), and environmental health (8 items); it also contains Quality of Life and general health items. Each item of the WHOQOL-BREF is scored on a 1 to 5 response scale, which is stipulated as a 5-point ordinal scale. The scores are then transformed linearly to a 0‐100 scale. Higher scores on the WHOQOL-BREF reflect a greater quality of life.

##### Self-Efficacy

The Harm Reduction Self-Efficacy Scale (HRSES) [80] is a 12-item measure that asks participants to indicate on a 0% to 100% scale regarding how confident they are that they would be able to resist drinking beyond their limit in a given situation. Higher scores on the HRSES reflect greater confidence to resist drinking beyond one’s limit.

##### Addiction Cycle Domains

The Addictions Neuroclinical Assessment has been used to represent domains of the addiction cycle and is assessed using a 12-item scale with 4 items for each domain [81]. The negative emotionality domain of the addiction cycle is characterized by temptation to drink in situations when the person is experiencing negative emotions. The Negative Emotionality Scale consists of 4 items scored on a scale from 1 (not at all tempted) to 5 (extremely tempted) to drink when experiencing negative emotions. Higher scores indicate a greater tendency to be tempted to drink in situations characterized by negative emotions. The incentive salience domain of the addiction cycle is characterized by temptation to drink in situations when the person is experiencing rewarding or social pressure to drink. The Incentive Salience Scale consists of 4 items scored on a scale from 1 (not at all tempted) to 5 (extremely tempted) to drink when experiencing rewarding, craving, or social situations. Higher scores indicate a greater tendency to be tempted to drink in situations characterized by reward, craving, and social pressure. The executive function domain of the addiction cycle is characterized by loss of control over drinking. The Executive Scale consists of 1 binary (yes or no) item (“After taking one or two drinks, can you usually stop?") and 4 items scored on a scale from 1 (indicating more control over drinking) to 5 (indicating loss of control over drinking). Higher scores indicate more impaired executive function and greater loss of control over drinking.

##### Purpose in Life

The Purpose in Life test [82,83] is a 20-item measure of experiencing meaning and purpose in one’s life, with each item assessed on a 1 (lack of meaning) to 7 (full of meaning) Likert-type scale. Higher scores indicate more meaning and purpose in life.

### Participant Timeline

From randomization, client participants are enrolled in the study for a total duration of 36 months. In total, there are 7 assessment sessions (enrollment, 6-, 12-, 18-, 24-, 30-, and 36-month follow-ups). The enrollment session lasts approximately 120 minutes (to allow for consent and interviewing), while the other assessment sessions last a maximum of 60 minutes. provides an overview of the schedule of assessment events for an individual study participant (Table 2).

**Table 2.** Summary of study activities timeline.

Activity	Participant timeline
Phone or online screening	—^a^
Start of study participation: consent, randomization, and initial assessment (self-report measures, interview, and dried blood spot)	Month 0
Orientation session for randomized condition	Month 0
Attend MBRP^b^ groups or online mutual support groups	Month 0-36
6-month assessment	Month 6
12-month assessment	Month 12
18-month assessment	Month 18
24-month assessment	Month 24
30-month assessment	Month 30
End of study participation: 36-month assessment (self-report measures, interview, and dried blood spot card)	Month 36

a Not applicable.

b MBRP: mindfulness-based relapse prevention.

### Sample Size Considerations

Based on prior experience modeling outcomes over time and examining differences between MBRP and comparison groups, we are interested in powering the study to detect a small effect size difference between MBRP and referral to online mutual support in supporting recovery (primary outcome). We estimate power based on our prior efficacy trial [44] in which the difference at 12 months posttreatment on cessation of heavy drinking between MBRP and treatment as usual was a Cohen h=0.36. With a 2-tailed α of .05 and 430 participants (215 per group), we anticipate power greater than .90 to detect a similar effect size. To further ascertain power for the proposed set of analyses, Monte Carlo simulation studies were conducted to estimate power for testing the secondary outcomes given the proposed modeling approaches and likely parameter estimates. Power is greater than .90 for examining the effects of MBRP versus referral to mutual support on secondary outcomes over time. Given the use of modern missing data handling techniques [84,85] and an intent-to-treat design, we do not need to adjust sample size for potential attrition in the trial, although we aim to have 80% retention across the trial.

### Recruitment

Participants are recruited from web-based advertisements, posting of recruitment flyers throughout communities in targeted locations (including at treatment agencies and oft-visited locations near treatment agencies), online and social media recruitment sites, and links for recruitment on the homepage of study investigators. Recruitment materials include a QR code that is linked to a digital version (web page) of the participant recruitment information and online screening form. The website does not ask for any personal information. Participants can click a link from the web page to access a secure REDCap survey where they can provide their contact information (ie, name, email, and phone number) and complete an online screening form. Additionally, recruitment is completed via BuildClinical, a data-driven platform that helps academic researchers recruit participants for research studies more efficiently using social media, software, and machine learning. BuildClinical uses study-specific advertisements to engage participants on digital platforms such as Facebook (Meta Platforms, Inc), Google (Google LLC), WebMD (WebMD Health Corp), etc and redirect them to a study-specific landing page should they click it. On the landing page, the person can complete an online prescreen questionnaire that gets routed into BuildClinical’s platform. BuildClinical’s Secure Socket Layer encrypts all input information and keeps information private and HIPAA-compliant. Additional screening after the BuildClinical screen is then completed by the study staff, and eligible participants are emailed or texted an additional online screening form via a secure REDCap survey.

### Assignment of Interventions

Randomization is completed via the REDCap randomization module with an allocation sequence generated using a random number generator in Excel and with blocking on AUD symptoms and sex assigned at birth. The allocation sequence is uploaded into the randomization module of REDCap by unblinded study team members. A postdoctoral fellow generated the allocation sequence and uploaded it into the randomization module of REDCap, which then randomized participants to intervention conditions. Study staff who are blinded to the intervention condition enroll the participants.

Given the design of the study and use of behavioral interventions, only some members of the team could be blinded. Study participants are not blinded, given that they knew which continuing care program they were receiving. MBRP group facilitators and supervisors, as well as those delivering the orientation sessions, could also not be blinded. All outcome assessors, the study PI, and data analysts are blinded. The study PI is only unblinded on a case-by-case basis by an unblinded study team investigator in the case of a severe adverse event that involves participant allocation in the MBRP intervention condition and reporting to IRB and funding agencies regarding participation in the MBRP intervention.

### Data Collection and Management

Study data are collected and managed using REDCap [86,87], a secure, web-based software platform designed to support data capture for research studies hosted at the UNM CASAA. Data quality is promoted through careful programming to limit out-of-range responses. Study staff are continuously monitored to ensure data quality and protocol and regulatory adherence using applicable national and local regulations and standards.

Participants complete self-report questionnaire measures at baseline and every 6 months for 3 years following baseline. The measures were selected to assess specific constructs and were selected for previous evidence of producing reliable and valid scores, particularly in studies of AUD intervention programs. All measures and citations are provided in Table 3.

In addition to the self-report measures, we also collect physical mailing addresses in order to measure alcohol use via dried blood spot cards at baseline and the 3-year follow-up. A dried blood spot card or collection device is mailed to participants at baseline and month 36 to assess phosphatidylethanol (PEth), a biological marker that is sensitive to alcohol use and can be feasibly collected via mail [88,89]. The dried blood spot card collection kit, offered by the US Drug Testing Laboratories, requires a specimen amount that is 5 dried blood spots from a finger puncture or 5 mL of blood from a standard blood draw using an anticoagulation tube collection. Standard collection supplies provided include 2 lancets, 2 non–ethanol-based alcohol pads, gauze, a collection card, and the dried blood spot drying box. Returning the dried blood spot card is incentivized, and considerable effort is devoted to obtaining PEth samples from all participants. The dried blood spot cards are identified only via participant ID, shipped to the US Drug Testing Laboratories, who then email the study team with PEth values, which are recorded to the study REDCap database by trained research staff. No specimens are stored for future use, and no third parties have access to identifiable participant data.

MBRP facilitators are administered the MBRP self-report assessments listed in Table 3 above, including the Five Facet Mindfulness Questionnaire - Short Form, the Self-Compassion Scale - Short Form, the MBRP Practice Questionnaire, and the Applied Mindfulness Process Scale. MBRP facilitators also complete a measure of mindfulness meditation practice and retreat experience.

Process evaluation measures are guided by the RE-AIM (Reach, Effectiveness, Adoption, Implementation, Maintenance) model [90,91], which describes public health impact related to implementation research: (1) Reach: how many participants are impacted (eg, number of participants that engage in the MBRP program); (2) Effectiveness, such as rates of recovery and improvements in functioning among those who attend MBRP groups; (3) Adoption: continued engagement in offering the MBRP groups among the community clinician facilitators; (4) Implementation: extent to which the MBRP groups are attended and fidelity to the MBRP manual; and (5) Maintenance: the extent to which participants would continue attending MBRP groups and the extent to which community clinician facilitators would continue to offer the MBRP groups. We use a mixed methods framework. The quantitative metrics are gathered throughout the study. The qualitative interview guide is based on RE-AIM QUEST (Qualitative Evaluation for Systematic Translation) [90], tailored for MBRP. Qualitative interviews are conducted one-on-one with trained assessors via video calls. Facilitator participants and client participants are asked about their experiences with mindfulness generally, the THRIVE MBRP groups specifically, the THRIVE mobile app, and questions to assess potential facilitators and barriers to implementation of telehealth-delivered MBRP groups similar to those in THRIVE.

**Table 3.** Constructs measured and specific assessments repeated every 6 months for 3 years.

Constructs	Assessment measures
Alcohol and drug use	Timeline Followback [67], alcohol and drug use past 6 months.
AUD^a^ severity	Alcohol Dependence Scale [92]
Diagnosis	*DSM-5^b^* AUD symptoms assessed by interview [93]
Alcohol problems	PROMIS^c^ alcohol consequences [75]
Craving	Penn Alcohol Craving Scale [79]
Psychosocial functioning	Psychosocial Functioning Inventory [94] WHOQOL-BREF^d^ [68] Purpose in Life [95]
Physical and mental health	PROMIS PROPr^e^ (functioning, sleep, pain, etc) [76], and PROMIS Meaning and Purpose measure [78]
Economic resources, housing, and food security	Employment status, income, transportation, housing, and food security
Social support	Social Support Questionnaire [96] Important People and Activities [97]
Incentive Salience items from AASE^f^ [98]	When I dream about taking a drink (AASE) When offered a drink in a social situation (AASE) When I feel physical need or craving for a drink (AASE) When I see others drinking (AASE)
Negative Emotionality items from AASE [98] and AUI^g^ [99]	When I am feeling depressed (AASE) When I am worried (AASE) When I feel angry inside (AASE) I start drinking to get over being depressed (AUI)
Executive Functioning items from the AASE [98], ADS^h^ [92], and OCDS^i^ [100]	Able to control amount you drink (ADS) Can usually stop after 1 or 2 drinks (ADS) Lose control over what you do when drinking (ADS) Success in stopping thoughts of drinking (OCDS) Effort to resist drinking (OCDS) Strong drive to consume alcohol (OCDS) Control over drinking (OCDS)
Treatment history, recovery resources, and recovery capital	Treatment and mutual-help history and goals from Recovery Resource Use Brief Assessment of Recovery Capital [101]
Self-efficacy	Substance Use Moderation Self-Efficacy Scale [102]
MBRP^j^ measures	Modified Group Cohesion Scale [103] Five Facet Mindfulness Questionnaire [104] Self-Compassion Scale [105] MBRP Practice Questionnaire Applied Mindfulness Process Scale [106]
Mutual-help measure	Multidimensional Measure of Mutual-Help Activity [107]

a AUD: alcohol use disorder.

b *DSM-5*: *Diagnostic and Statistical Manual for Mental Disorders *(Fifth Edition).

c PROMIS: Patient-Reported Outcomes Measurement Information System.

d WHOQOL-BREF: World Health Organization Quality of Life–Brief Version.

e PROPr: Patient-Reported Outcomes Measurement Information System Preference.

f AASE: Alcohol Abstinence Self-Efficacy Scale.

g AUI: Alcohol Use Inventory.

h ADS: Alcohol Dependence Scale.

i OCDS: Obsessive Compulsive Drinking Scale.

j MBRP: mindfulness-based relapse prevention.

### Retention

The investigative team is highly experienced in the conduct of clinical trials, and CASAA’s Program Evaluation Services staff routinely achieve follow-up rates at 1-year posttreatment of 85%‐90%, and they achieved 85% in the 3-year follow-up to Project MATCH [108]. These follow-up rates are achieved by creating a supportive atmosphere for research participants, through extensive participant tracking methods, and through compensation for assessments that is roughly equal to $25‐40/hour, by providing increasing compensation over time, and providing bonuses for completing at least 4 of the 6 follow-up assessment visits. Additional CASAA study staff send thank you cards, birthday cards, holiday cards, and study-branded materials (eg, pens, magnets, and study timeline) to participants to encourage retention. All outcome data are collected regardless of discontinuation or deviation from intervention protocols.

### Data Management

Data collection and accurate documentation are the responsibility of the study staff under the supervision of the study PI. Source documents and laboratory reports are reviewed by the study team and data entry staff, who will ensure that they are accurate and complete. The data are collected and maintained by experienced study staff who are trained in the use of clinical and research assessments using Good Clinical Practice tools. Study staff receive confidentiality and human participants’ protection training. All data are used for research purposes only and will not be revealed without the participants’ consent, and all entered data are checked first manually by another study staff member for correct entry and then statistically for correct form and range. Electronic data are stored in a database on a secure, password-protected server that is regularly backed up, and data collection forms are entered into REDCap. Data collection forms have been used in our previous studies and are retested prior to use. Data are monitored on a daily basis during the baseline period to track response rates and assess data quality. Protocol training is completed prior to enrollment. The research team is trained on best practices to follow the protocol procedures, consent, data collection, data entry, and query resolution. Scoring syntax that was developed for our prior studies is used to produce summary variables, and standard procedures are used for dealing with missing data.

### Statistical Methods

#### Primary Outcome Analysis

We use a generalized linear mixed model (GLMM) that can accommodate a binary outcome (recovery vs not recovery as defined by the NIAAA definition [17]) and random effects that correct for the nonindependence of repeated measures in longitudinal data to assess within-person change over time. The recovery binary variable is created using the 3 indicators included in the definition at each time point. AUD remission is based on not endorsing criteria for AUD (other than craving which may persist even during abstinence), cessation of heavy drinking is defined by no self-reported heavy drinking days, and improvements in functioning and well-being as measured by higher score (from baseline) on the WHOQOL-BREF [68] domain scores (each of 26 items scored from 1 to 5 on a response scale, which are then transformed linearly to a 0‐100 scale) and the 36-item Short Form Health Survey [69] Mental Health Component Score (scored on a 0‐100 scale). The analyses consist of estimating a GLMM with fixed effects of baseline sociodemographics and covariates used in randomization (sex and AUD severity), fixed effects of random assignment to MBRP versus online mutual support referral, and random effects of time.

#### Secondary Outcome Analyses

We use a GLMM that can accommodate binary secondary outcomes (achieving at least a 1-level reduction and achieving at least a 2-level reduction in WHO risk levels) and a general linear mixed model that can accommodate continuous secondary outcome measures. In all analyses, we include random effects that correct for the nonindependence of repeated measures in longitudinal data to assess within-person change in secondary outcomes over time. The analyses consist of estimating a mixed model with fixed effects of baseline sociodemographics and covariates used in randomization (sex and AUD severity), fixed effects of random assignment to MBRP versus online mutual support referral, and random effects of time.

#### Mechanisms of Change Analyses

We use GLMM and GLM to examine associations between addiction cycle domains and the primary and secondary outcomes. We hypothesize improvements in executive functioning, self-efficacy, and recovery capital, and reductions in incentive salience and negative emotionality over time predict a higher likelihood of recovery and greater improvement in secondary outcomes at the 3-year follow-up. To test whether MBRP versus online mutual support uniquely impacts mechanisms of behavior change, we use latent growth mediation analyses with randomized condition (independent variable), addiction cycle domains, and self-efficacy over time (mediators), and the recovery outcome or secondary outcomes, as we have done in prior work [109,110]. The product of coefficients approach is used to assess the statistical significance with bootstrapping to obtain 95% CIs of the mediated effects [111]. Sensitivity analyses will also be conducted to estimate the robustness of the mediating effects to the no-omitted confounder assumption [56]. All models are reestimated with the number of sessions of MBRP or online mutual support groups attended as the independent variable.

#### Interim and Subgroup Analyses

No interim analyses are conducted. As the literature suggests possible differences in outcomes based on sex, gender, race, and ethnicity [112-114], we examine sex, gender, race, and ethnicity as moderators of group effects. We also examine whether there are facilitator effects (eg, effects of a particular MBRP facilitator) by incorporating the facilitator as a random effect in the mixed effects models. We also test whether addiction cycle domains moderate the association between MBRP and primary and secondary outcomes.

#### Analysis Approach and Missing Data

Primary analyses are performed under the modified intent-to-treat (mITT) principle, whereby each eligible participant is analyzed according to the intervention group to which they were assigned at the time of randomization. Moreover, the mITT sample excludes participants who never attended the orientation session or those who were deemed ineligible after randomization. The rationale for using the mITT sample in primary analyses is to address the fact that some participants, in either randomization group, do not initiate treatment. The ITT estimates the effect of the assigned treatment and is biased toward the null to estimate the effect of the intervention (in the absence of noninitiation). The mITT estimator is appropriate because (1) noninitiation is identifiable in each group and (2) it is reasonable to assume that if someone does not initiate treatment in one arm, they would also not initiate treatment in the other arm; in fact, both arms begin with the same introductory orientation session.

Additionally, we conduct a per-protocol analysis that includes only participants who attend at least 2 sessions of the MBRP groups or correspondingly attended at least 2 mutual help group sessions. The per-protocol analysis excludes participants who did not attend at least 2 intervention sessions or those who were deemed ineligible after randomization.

Multiple imputation is used to accommodate missing outcome data [115]. We test missing data assumptions using sensitivity analyses [85,116]. We generate 100 imputed datasets, fit the primary analysis model to each imputed dataset, and combine the results by using Rubin rules.

#### Data Sharing

Sharing of data is germane to the advancement of science and medicine for the public good. This study will comply with all applicable NIH Data Sharing Policies. The investigators comply with all NIAAA Data Archives policies established during the project period. This includes compliance with the NIAAA central data platform requirements and timelines developed through the NIAAA Data Share. Institutions that receive Data and/or Materials from this award for performance of activities under this award are required to use the Data and/or Materials only as outlined by the NIAAA Data Share in a manner that is consistent with applicable state and federal laws and regulations, including any informed consent requirements and the terms of the institution’s NIH funding, including NOT-OD-17‐109 and 42 U.S.C. 241(d). A releasable database is produced and is completely deidentified in accordance with the definitions provided in HIPAA. Namely, all identifiers are recoded in a manner that will make it impossible to deduce or impute the specific identity of any participant. The database does not contain any institutional identifiers.

#### Oversight and Monitoring

KW is the PI of the trial. She is a licensed clinical psychologist and quantitative methodologist who has devoted her career to studying mindfulness-based interventions for AUD and other substance use disorders, and who has spent the last 5 years studying nonabstinent recovery and addiction cycle domains as predictors of recovery outcomes in AUD clinical trials. Co-investigator, Dr Matthew R Pearson, has served as lead or co-lead on several recent analyses examining nonabstinent recovery and will assist with all analyses. Dr Pearson also brings considerable expertise in nationwide recruitment [117] and recruitment of individuals in early AUD recovery from InTheRooms (R01AA027508). Co-investigator, MK, is a licensed clinical psychologist with experience implementing MBRP in clinical settings, which included developing and adapting MBRP programming in clinical settings where it was previously unavailable. MK is unblinded and oversees day-to-day operations of the study, including data monitoring and participant safety reportable to the PI. Co-investigator, FJS, brings expertise in delivering MBRP via Zoom as one of the therapists in a recently completed clinical trial [118] and has recent experience with nationwide recruitment of individuals engaged in harm reduction groups [102]. ARV, co-investigator, brings expertise in community-based participatory research and qualitative interviews [119-121].

Program Evaluation Services, directed by RC, and her staff AM, RQ, and RO, provide the day-to-day oversight of the trial, in partnership with the research coordinator, DSO.

The consultants, SB, CRR, and CV, are experts in the implementation of MBRP. SB developed the MBRP program and has been training and supervising practitioners in delivering MBRP for the past 15 years. CRR developed the first rolling group version of MBRP [62] and has also investigated the similarities and differences between MBRP and mutual support programs [43]. CV was the PI on our recent pilot study examining MBRP via Zoom [59]. All investigators have expertise in substance use disorder treatment and research, and virtual research assessments and intervention delivery.

The study established a Data Safety Monitoring Board (DSMB) in the first year of the trial, composed of 3 established investigators external to the study. DSMB members are provided with annual reports of progress in the study, including information about screening (inclusion and rate of exclusion), demographics, and adverse events data. The committee uses these reports to decide whether we can continue recruitment, propose a protocol amendment, or stop recruitment for the trial. Severe adverse events are reported immediately to the DSMB. The DSMB members are then asked to make a recommendation regarding continuing the study, scheduling a formal meeting to discuss the event, or immediately stopping the study. If any members of the DSMB request a meeting, then the PI schedules a formal meeting within 1 week of the request. At the meeting, the DSMB holds a vote on whether the study should (1) continue unchanged, (2) make a protocol amendment, or (3) stop recruitment. If any DSMB member requests a protocol amendment or discontinuation of the study, the PI informs the IRB and funding agency immediately.

It is the responsibility of the PI, KW, to obtain and maintain approval of all procedures from the UNM. On a regular basis, the research team reports on the procedures being used in the protocol to ensure that all IRB-approved procedures are being followed. Any adverse events are reported to the PI immediately. Serious adverse events and unanticipated problems are reported to the local IRB and funding agency (NIAAA) project officer within 48 hours. In addition, the study PI prepares a summary of the DSMB report to NIAAA annually as part of the progress report. There are no plans for auditing trial conduct, although the UNM IRB could decide to audit the trial at any time. Protocol modifications are reported to the local IRB, DSMB, and NIH ClinicalTrial registry.

#### Dissemination

We registered the trial with ClinicalTrials.gov and provided an informational website through the CASAA. Informed consent documents for the trial include a specific statement relating to the posting of clinical trials information on ClinicalTrials.gov. All clinical trials registration and reporting of results occur in compliance with the policies of the UNM and the NIH.

We expect to present preliminary results at scientific meetings, including the Collaborative Perspectives of Addiction, Research Society on Alcohol, and Society for Implementation Research Collaboration. All results are disseminated through published manuscripts and presentations at scientific meetings. After the primary results have been published, the database will be cleaned and purged of identifiers and made available to researchers for secondary analyses via the NIAAA Data Archive.

## Results

Funding for this study was awarded by the NIAAA (R01AA031159, PI: Witkiewitz) in August 2023. The version date for the latest protocol is December 22, 2025, and this is version 3.0 of the Protocol. Protocol changes included adding measures, adding an initial screening form online (vs via phone), increasing sample size, and adding procedures for participants to be recruited by other researchers. Recruitment began on September 13, 2023, and the last recruited participant was randomized on March 20, 2025. We randomized 470 individuals, of whom 432 completed orientation sessions, just going past the trial target of n=430 (100.5% recruited). The 6-month data collection has also been completed for all participants, and 369 completed the 6-month follow-up, 34 withdrew from the study prior to the 6-month follow-up; thus, the follow-up completion rate among those who continued in the study was 369/436 (86%) and the overall follow-up rate for all randomized participants was 369 out of 470 (78.5%).

## Discussion

### Principal Findings

This paper describes a hybrid type 1 effectiveness-implementation design to prospectively test the effectiveness of MBRP and to identify barriers and facilitators of group participation to inform future implementation of MBRP as continuing care. We hypothesize that MBRP may support longer-term recovery from AUD via a broader focus on whole-person functioning and by targeting addiction domain cycles.

### Limitations and Strengths

There are several design considerations that should be highlighted. First, we chose a 2-group design and will not randomize to different kinds of mutual support (eg, SMART Recovery vs AA). In our initial power analyses, we found that including another randomization group would more than double the required sample size. Second, we opted not to directly recruit from specific treatment programs and to partner with specific AUD treatment programs, and to also include those who make changes in the absence of treatment. We view this as a limitation and a strength. Although we collect information about the amount and type of treatment received (via the treatment history form), we are relying on self-report and we are not able to objectively assess whether specific AUD treatments, as actually delivered by the treatment programs, are associated with recovery outcomes. This design decision is also a strength in that the findings may generalize to a number of different treatment programs or those who make changes without treatment. Thus, the study assesses the effectiveness of MBRP as continuing care regardless of how people make initial changes in their drinking (through treatment or self-change). Finally, self-reported drinking and drug use measured retrospectively via the Timeline Followback has limitations [122]. It would be impossible to quantify pre-baseline levels of drinking and drug use with any other method and quantifying within-person change in drinking and drug use is critical for our aims and hypotheses. We have included PEth to biochemically assess drinking at baseline and 3-year follow-up.

### Future Directions

If our hypotheses are supported, findings would set the stage for several avenues for future research and widespread dissemination, including the communication of findings within conference presentations and peer-reviewed manuscripts, as well as making the MBRP via Zoom manual, orientation materials, participant worksheets, and facilitator training materials publicly available. This would serve to expand continuing care options and provide an alternative for individuals who are seeking non–abstinence-based recovery support.

## Supplementary material

10.2196/92198 Peer Review Report 1 Peer review report by the AA-3 - Clinical, Treatment and Health Services Research Study Section, National Institute on Alcohol Abuse and Alcoholism Initial Review Group (National Institutes of Health, USA).

## Abbreviations

AA: Alcoholics Anonymous
AUD: alcohol use disorder
CASAA: Center on Alcohol, Substance Use, and Addictions
CoC: Certificate of Confidentiality
*DSM-5*: *Diagnostic and Statistical Manual for Mental Disorders* (Fifth Edition)
DSMB: Data Safety Monitoring Board
GLMM: generalized linear mixed model
HHS: Department of Health and Human Services
HIPAA: Health Insurance Portability and Accountability Act
HRSES: Harm Reduction Self-Efficacy Scale
IRB: Institutional Review Board
MBRP: mindfulness-based relapse prevention
MBRP-AC: Mindfulness-Based Relapse Prevention Adherence and Competence Scale
mITT: modified intent-to-treat
NIAAA: National Institute on Alcohol Abuse and Alcoholism
NIH: National Institutes of Health
PEth: phosphatidylethanol
PI: principal investigator
PROMIS: Patient-Reported Outcomes Measurement Information System
PROPr: Patient-Reported Outcomes Measurement Information System Preference
QUEST: Qualitative Evaluation for Systematic Translation
RE-AIM: Reach, Effectiveness, Adoption, Implementation, Maintenance
REDCap: Research Electronic Data Capture
SF-36: 36-item Short Form Health Survey
SOBER: Stop-Observe-Breathe-Expand-Respond
THRIVE: Telehealth Resources for Individualized Recovery
UNM: University of New Mexico
WHO: World Health Organization
WHOQOL-BREF: World Health Organization Quality of Life–Brief Version

## References

[1] Glantz MD, Bharat C, Degenhardt L (2020). The epidemiology of alcohol use disorders cross-nationally: findings from the World Mental Health Surveys. Addict Behav.

[2] Sacks JJ, Gonzales KR, Bouchery EE, Tomedi LE, Brewer RD (2015). 2010 national and state costs of excessive alcohol consumption. Am J Prev Med.

[3] Esser MB, Leung G, Sherk A (2022). Estimated deaths attributable to excessive alcohol use among US adults aged 20 to 64 years, 2015 to 2019. JAMA Netw Open.

[4] Babor TF, Casswell S, Graham K (2022). Alcohol: no ordinary commodity-a summary of the third edition. Addiction.

[5] Rehm J, Gmel G, Sempos CT, Trevisan M (2003). Alcohol-related morbidity and mortality. Alcohol Res Health.

[6] Chisholm D, Moro D, Bertram M (2018). Are the “Best Buys” for alcohol control still valid? An update on the comparative cost-effectiveness of alcohol control strategies at the global level. J Stud Alcohol Drugs.

[7] Bazzi AR, Syvertsen JL, Rolón ML (2016). Social and structural challenges to drug cessation among couples in Northern Mexico: implications for drug treatment in underserved communities. J Subst Abuse Treat.

[8] Pullen E, Oser C (2014). Barriers to substance abuse treatment in rural and urban communities: counselor perspectives. Subst Use Misuse.

[9] (2009). Office of Applied Studies, Results from the 2008 National Survey on Drug Use and Health: National Findings (NSDUH Series H-36, HHS Publication NoSMA 09-4443.

[10] Witkiewitz K, Litten RZ, Leggio L (2019). Advances in the science and treatment of alcohol use disorder. Sci Adv.

[11] (2021). Substance abuse and mental health services administration, “Key substance use and mental health indicators in the United States: results from the 2020 National Survey on Drug Use and Health (HHS publication no.PEP21-07-01-003, NSDUH series h-56). https://www.samhsa.gov/data/report/2020-nsduh-annual-national-report.

[12] Henssler J, Müller M, Carreira H, Bschor T, Heinz A, Baethge C (2021). Controlled drinking-non-abstinent versus abstinent treatment goals in alcohol use disorder: a systematic review, meta-analysis and meta-regression. Addiction.

[13] Hasin DS, Wall M, Witkiewitz K (2017). Change in non-abstinent WHO drinking risk levels and alcohol dependence: a 3 year follow-up study in the US general population. Lancet Psychiatry.

[14] Knox J, Wall M, Witkiewitz K (2019). Reduction in non-abstinent World Health Organization (WHO) drinking risk levels and drug use disorders: 3-year follow-up results in the US general population. Drug Alcohol Depend.

[15] Witkiewitz K, Hallgren KA, Kranzler HR (2017). Clinical validation of reduced alcohol consumption after treatment for alcohol dependence using the World Health Organization risk drinking levels. Alcohol Clin Exp Res.

[16] Witkiewitz K, Wilson AD, Roos CR (2021). Can individuals with alcohol use disorder sustain non-abstinent recovery? Non-abstinent outcomes 10 years after alcohol use disorder treatment. J Addict Med.

[17] Hagman BT, Falk D, Litten R, Koob GF (2022). Defining recovery from alcohol use disorder: development of an NIAAA research definition. Am J Psychiatry.

[18] (2013). Diagnostic and Statistical Manual of Mental Disorders, Fifth Edition (DSM-5).

[19] Witkiewitz K, Tucker JA (2020). Abstinence not required: expanding the definition of recovery from alcohol use disorder. Alcohol Clin Exp Res.

[20] Witkiewitz K, Montes KS, Schwebel FJ, Tucker JA (2020). What is recovery? A narrative review of definitions of recovery from alcohol use disorder. Alcohol Res.

[21] Koob GF, Volkow ND (2016). Neurobiology of addiction: a neurocircuitry analysis. Lancet Psychiatry.

[22] Koob GF, Volkow ND (2010). Neurocircuitry of addiction. Neuropsychopharmacology.

[23] Litten RZ, Ryan ML, Falk DE, Reilly M, Fertig JB, Koob GF (2015). Heterogeneity of alcohol use disorder: understanding mechanisms to advance personalized treatment. Alcohol Clin Exp Res.

[24] Marlatt GA, Parks GA, Witkiewitz K (2002). Clinical guidelines for implementing relapse prevention therapy.

[25] McKay JR (2021). Impact of continuing care on recovery from substance use disorder. Alcohol Res.

[26] McKay JR, Hiller-Sturmhofel S (2011). Treating alcoholism as a chronic disease: approaches to long-term continuing care. Alcohol Res Health.

[27] Kelly JF, Humphreys K, Ferri M (2020). Alcoholics Anonymous and other 12-step programs for alcohol use disorder. Cochrane Database Syst Rev.

[28] Tonigan JS, Pearson MR, Magill M, Hagler KJ (2018). AA attendance and abstinence for dually diagnosed patients: a meta-analytic review. Addiction.

[29] Tonigan JS, Martinez-Papponi B, Hagler KJ, Greenfield BL, Venner KL (2013). Longitudinal study of urban American Indian 12-step attendance, attrition, and outcome. J Stud Alcohol Drugs.

[30] Owen PL, Slaymaker V, Tonigan JS (2003). Participation in Alcoholics Anonymous: intended and unintended change mechanisms. Alcohol Clin Exp Res.

[31] Tonigan JS, Rynes KN, McCrady BS (2013). Spirituality as a change mechanism in 12-step programs: a replication, extension, and refinement. Subst Use Misuse.

[32] Kelly JF, Stout RL, Magill M, Tonigan JS, Pagano ME (2011). Spirituality in recovery: a lagged mediational analysis of alcoholics anonymous’ principal theoretical mechanism of behavior change. Alcohol Clin Exp Res.

[33] Kelly JF (2017). Is Alcoholics Anonymous religious, spiritual, neither? Findings from 25 years of mechanisms of behavior change research. Addiction.

[34] Kelly JF, Stout RL, Magill M, Tonigan JS, Pagano ME (2010). Mechanisms of behavior change in Alcoholics Anonymous: does Alcoholics Anonymous lead to better alcohol use outcomes by reducing depression symptoms?. Addiction.

[35] Zemore SE, Kaskutas LA (2004). Helping, spirituality and Alcoholics Anonymous in recovery. J Stud Alcohol.

[36] Kelly JF, Abry AW (2021). Leave the past behind by recognizing the effectiveness and cost-effectiveness of 12-step facilitation and Alcoholics Anonymous. Alcohol Alcohol.

[37] Venner KL, Greenfield BL, Vicuña B, Muñoz R, Bhatt S, O’Keefe V (2012). “I’m not one of them”: barriers to help-seeking among American Indians with alcohol dependence. Cultur Divers Ethnic Minor Psychol.

[38] Kaskutas LA (1994). What do women get out of self-help? Their reasons for attending Women for Sobriety and Alcoholics Anonymous. J Subst Abuse Treat.

[39] Vederhus JK, Høie M, Birkeland B (2020). One size doesn’t fit all: a thematic analysis of interviews with people who have stopped participating in Narcotics Anonymous in Norway. Addict Sci Clin Pract.

[40] Walters GD (2002). Twelve reasons why we need to find alternatives to Alcoholics Anonymous. Addict Disord Their Treat.

[41] Sally Glassman H, Rhodes P, Buus N (2022). Exiting Alcoholics Anonymous disappointed: a qualitative analysis of the experiences of ex-members of AA. Health (London).

[42] Kelly JF, Hoeppner B, Stout RL, Pagano M (2012). Determining the relative importance of the mechanisms of behavior change within Alcoholics Anonymous: a multiple mediator analysis. Addiction.

[43] Marcovitz DE, McHugh KR, Roos C, West JJ, Kelly J (2020). Overlapping mechanisms of recovery between professional psychotherapies and Alcoholics Anonymous. J Addict Med.

[44] Bowen S, Witkiewitz K, Clifasefi SL (2014). Relative efficacy of mindfulness-based relapse prevention, standard relapse prevention, and treatment as usual for substance use disorders: a randomized clinical trial. JAMA Psychiatry.

[45] Witkiewitz K, Warner K, Sully B (2014). Randomized trial comparing mindfulness-based relapse prevention with relapse prevention for women offenders at a residential addiction treatment center. Subst Use Misuse.

[46] Bowen S, Chawla N, Collins SE (2009). Mindfulness-based relapse prevention for substance use disorders: a pilot efficacy trial. Subst Abuse.

[47] Korecki JR, Schwebel FJ, Votaw VR, Witkiewitz K (2020). Mindfulness-based programs for substance use disorders: a systematic review of manualized treatments. Subst Abuse Treat Prev Policy.

[48] Grant S, Colaiaco B, Motala A (2017). Mindfulness-based relapse prevention for substance use disorders: a systematic review and meta-analysis. J Addict Med.

[49] Bowen S, Chawla N, Grow J, Alan MG (2021). Mindfulness-Based Relapse Prevention for Addictive Behaviors: A Clinician’s Guide.

[50] Witkiewitz K, Marlatt GA (2004). Relapse prevention for alcohol and drug problems: that was Zen, this is Tao. Am Psychol.

[51] Zemestani M, Ottaviani C (2016). Effectiveness of mindfulness-based relapse prevention for co-occurring substance use and depression disorders. Mindfulness (N Y).

[52] Davis JP, Berry D, Dumas TM (2018). Substance use outcomes for mindfulness based relapse prevention are partially mediated by reductions in stress: Results from a randomized trial. J Subst Abuse Treat.

[53] Yaghubi M, Zargar F (2018). Effectiveness of mindfulness-based relapse prevention on quality of life and craving in methadone-treated patients: a randomized clinical trial. Addict Healt.

[54] Brown DR, Jackson TCJ, Claus ED (2020). Decreases in the late positive potential to alcohol images among alcohol treatment seekers following mindfulness-based relapse prevention. Alcohol Alcohol.

[55] Witkiewitz K, Bowen S, Douglas H, Hsu S (2013). Mindfulness-based relapse prevention for substance craving. Addict Behav.

[56] Hsiao YY, Tofighi D, Kruger ES, Lee Van Horn M, MacKinnon DP, Witkiewitz K (2019). The (lack of) replication of self-reported mindfulness as a mechanism of change in mindfulness-based relapse prevention for substance use disorders. Mindfulness (N Y).

[57] Witkiewitz K, Bowen S (2010). Depression, craving, and substance use following a randomized trial of mindfulness-based relapse prevention. J Consult Clin Psychol.

[58] Vinci C, Sutton SK, Yang MJ (2023). Pilot randomized controlled trial of mindfulness-based relapse prevention vs cognitive behavioral therapy for smoking and alcohol use. Drug Alcohol Depend.

[59] Vinci C, Hemenway M, Baban SS (2022). Transition to telehealth: challenges and benefits of conducting group-based smoking and alcohol treatment virtually. Contemp Clin Trials.

[60] Curran GM, Bauer M, Mittman B, Pyne JM, Stetler C (2012). Effectiveness-implementation hybrid designs: combining elements of clinical effectiveness and implementation research to enhance public health impact. Med Care.

[61] Witkiewitz K, Stein ER, Votaw VR (2019). Mindfulness‐based relapse prevention and transcranial direct current stimulation to reduce heavy drinking: a double‐blind Sham‐controlled randomized trial. Alcohol Clin Exp Res.

[62] Roos CR, Kirouac M, Stein E, Wilson AD, Bowen S, Witkiewitz K (2019). An open trial of rolling admission mindfulness-based relapse prevention (rolling MBRP): feasibility, acceptability, dose-response relations, and mechanisms. Mindfulness (N Y).

[63] Caetano R, Clark CL, Greenfield TK (1998). Prevalence, trends, and incidence of alcohol withdrawal symptoms: analysis of general population and clinical samples. Alcohol Health Res World.

[64] Miller WR, Rollnick S (2013). Motivational Interviewing: Helping People Change.

[65] Chawla N, Collin S, Bowen S (2010). The mindfulness-based relapse prevention adherence and competence scale: development, interrater reliability, and validity. Psychother Res.

[66] Hallgren KA, Matson TE, Oliver M (2022). Practical assessment of alcohol use disorder in routine primary care: performance of an Alcohol Symptom Checklist. J Gen Intern Med.

[67] Sobell LC, Sobell MB (1992). Timeline Follow-Back: a technique for assessing self-reported alcohol consumption.

[68] The Whoqol Group (1998). Development of the World Health Organization WHOQOL-BREF Quality of Life assessment. Psychol Med.

[69] Ware JE, Sherbourne CD (1992). The MOS 36-item short-form health survey (SF-36). I. Conceptual framework and item selection. Med Care.

[70] Witkiewitz K, Anton RF, O’Malley SS (2025). Reductions in World Health Organization risk drinking levels as a Primary efficacy end point for alcohol clinical trials: a review. JAMA Psychiatry.

[71] World Health Organization (2000). International Guide for Monitoring Alcohol Consumption and Related Harm.

[72] Cella D, Yount S, Rothrock N (2007). The Patient-Reported Outcomes Measurement Information System (PROMIS). Med Care.

[73] Cella D, Riley W, Stone A (2010). The Patient-Reported Outcomes Measurement Information System (PROMIS) developed and tested its first wave of adult self-reported health outcome item banks: 2005-2008. J Clin Epidemiol.

[74] Cella D, Choi SW, Condon DM (2019). PROMIS^®^ adult health profiles: efficient short-form measures of seven health domains. Value Health.

[75] Pilkonis PA, Yu L, Colditz J (2013). Item banks for alcohol use from the Patient-Reported Outcomes Measurement Information System (PROMIS®): Use, consequences, and expectancies. Drug Alcohol Depend.

[76] Dewitt B, Jalal H, Hanmer J (2020). Computing PROPr utility scores for PROMIS® profile instruments. Value Health.

[77] Dewitt B, Feeny D, Fischhoff B (2018). Estimation of a preference-based summary score for the Patient-Reported Outcomes Measurement Information System: The PROMIS^®^-Preference (PROPr) scoring system. Med Decis Making.

[78] Salsman JM, Schalet BD, Park CL (2020). Assessing meaning & purpose in life: development and validation of an item bank and short forms for the NIH PROMIS^®^. Qual Life Res.

[79] Flannery BA, Volpicelli JR, Pettinati HM (1999). Psychometric properties of the Penn Alcohol Craving Scale. Alcohol Clin Exp Res.

[80] Schwebel FJ, Richards DK, Pearson MR, Witkiewitz K (2024). Initial psychometric testing of the harm reduction self-efficacy scale. Addict Res Theory.

[81] Witkiewitz K, Stein ER, Votaw VR (2023). Constructs derived from the addiction cycle predict alcohol use disorder treatment outcomes and recovery 3 years following treatment. Psychol Addict Behav.

[82] Crumbaugh JC, Maholick LT (1964). An experimental study in existentialism: the psychometric approach to Frankl’s concept of noogenic neurosis. J Clin Psychol.

[83] Crumbaugh JC, Henrion R (1988). The PIL Test: administration, interpretation, uses theory and critique. Int Forum Logother.

[84] Hallgren KA, Witkiewitz K (2013). Missing data in alcohol clinical trials: a comparison of methods. Alcohol Clin Exp Res.

[85] Witkiewitz K, Bush T, Magnusson LB, Carlini BH, Zbikowski SM (2012). Trajectories of cigarettes per day during the course of telephone tobacco cessation counseling services: a comparison of missing data models. Nicotine Tob Res.

[86] Harris PA, Taylor R, Minor BL (2019). The REDCap consortium: building an international community of software platform partners. J Biomed Inform.

[87] Harris PA, Taylor R, Thielke R, Payne J, Gonzalez N, Conde JG (2009). Research Electronic Data Capture (REDCap)--a metadata-driven methodology and workflow process for providing translational research informatics support. J Biomed Inform.

[88] Hahn JA, Murnane PM, Vittinghoff E (2021). Factors associated with phosphatidylethanol (PEth) sensitivity for detecting unhealthy alcohol use: an individual patient data meta-analysis. Alcohol Clin Exp Res.

[89] Bakhireva LN, Shrestha S, Gutierrez HL, Berry M, Schmitt C, Sarangarm D (2016). Stability of phosphatidylethanol in dry blood spot cards. Alcohol Alcohol.

[90] Forman J, Heisler M, Damschroder LJ, Kaselitz E, Kerr EA (2017). Development and application of the RE-AIM QuEST mixed methods framework for program evaluation. Prev Med Rep.

[91] Glasgow RE, Estabrooks PE (2018). Pragmatic applications of RE-AIM for health care initiatives in community and clinical settings. Prev Chronic Dis.

[92] Skinner HA, Horn JL (1984). Alcohol Dependence Scale: User’s Guide.

[93] First MB, Williams JBW, Karg RS, Spitzer RL (2015). Structured clinical interview for DSM-5—research version (SCID-5 for DSM-5.

[94] Feragne MA, Longabaugh R, Stevenson JF (1983). The Psychosocial functioning inventory. Eval Health Prof.

[95] Crumbaugh JC (1977). The Seeking of Noetic Goals Test (SONG): a complementary scale to the Purpose in Life Test (PIL). J Clin Psychol.

[96] Sarason IG, Levine HM, Basham RB, Sarason BR (1983). Assessing social support: the Social Support Questionnaire. J Pers Soc Psychol.

[97] Clifford PR, Longabaugh R (1991). Manual for the Administration of the Important People and Activities Instrument.

[98] DiClemente CC, Carbonari JP, Montgomery RP, Hughes SO (1994). The Alcohol Abstinence Self-Efficacy Scale. J Stud Alcohol.

[99] Horn JL, Wanberg KW, Foster FM (1990). Guide to the Alcohol Use Inventory (AUI).

[100] Anton RF (2000). Obsessive-compulsive aspects of craving: development of the Obsessive Compulsive Drinking Scale. Addiction.

[101] Vilsaint CL, Kelly JF, Bergman BG, Groshkova T, Best D, White W (2017). Development and validation of a Brief Assessment of Recovery Capital (BARC-10) for alcohol and drug use disorder. Drug Alcohol Depend.

[102] Schwebel FJ, Orban DG (2023). Online support for all: examining participant characteristics, engagement, and perceived benefits of an online harm reduction, abstinence, and moderation focused support group for alcohol and other drugs. Psychol Addict Behav.

[103] Morgan DG, Kosteniuk J, Stewart N, O’Connell ME, Karunanayake C, Beever R (2014). The telehealth satisfaction scale: reliability, validity, and satisfaction with telehealth in a rural memory clinic population. Telemed J E Health.

[104] Gu J, Strauss C, Crane C (2016). Examining the factor structure of the 39-item and 15-item versions of the Five Facet Mindfulness Questionnaire before and after mindfulness-based cognitive therapy for people with recurrent depression. Psychol Assess.

[105] Raes F, Pommier E, Neff KD, Van Gucht D (2011). Construction and factorial validation of a short form of the Self-Compassion Scale. Clin Psychol Psychother.

[106] Li MJ, Black DS, Garland EL (2016). The Applied Mindfulness Process Scale (AMPS): a process measure for evaluating mindfulness-based interventions. Pers Individ Dif.

[107] Kelly JF, Urbanoski KA, Hoeppner BB, Slaymaker V (2011). Facilitating comprehensive assessment of 12-step experiences: a multidimensional measure of mutual-help activity. Alcohol Treat Q.

[108] Project MATCH Research Group (1998). Matching alcoholism treatments to client heterogeneity: project MATCH three‐year drinking outcomes. Alcohol Clin Exp Res.

[109] Moniz-Lewis DIK, Stein ER, Bowen S, Witkiewitz K (2022). Self-efficacy as a potential mechanism of behavior change in mindfulness-based relapse prevention. Mindfulness (N Y).

[110] Maisto SA, Roos CR, O’Sickey AJ (2015). The indirect effect of the therapeutic alliance and alcohol abstinence self-efficacy on alcohol use and alcohol-related problems in Project MATCH. Alcohol Clin Exp Res.

[111] MacKinnon DP (2008). Introduction to Statistical Mediation Analysis.

[112] Gilbert PA, Saathoff E, Russell AM, Brown G (2022). Gender differences in lifetime and current use of online support for recovery from alcohol use disorder. Alcohol Clin Exp Res.

[113] Holzhauer CG, Cucciare M, Epstein EE (2020). Sex and gender effects in recovery from alcohol use disorder. Alcohol Res.

[114] Zemore SE, Gilbert PA, Pinedo M, Tsutsumi S, McGeough B, Dickerson DL (2021). Racial/ethnic disparities in mutual help group participation for substance use problems. Alcohol Res.

[115] Hallgren KA, Witkiewitz K, Kranzler HR (2016). Missing data in alcohol clinical trials with binary outcomes. Alcohol Clin Exp Res.

[116] Enders CK (2011). Missing not at random models for latent growth curve analyses. Psychol Methods.

[117] Pearson MR, Liese BS, Dvorak RD, Marijuana Outcomes Study Team (2017). College student marijuana involvement: perceptions, use, and consequences across 11 college campuses. Addict Behav.

[118] McCrady BS, Claus E, Witkiewitz K, Shiver A, Swartz M, Chávez R (2024). Neurocognitive and neurobehavioral mechanisms of change following psychological treatment for alcohol use disorder. Contemp Clin Trials.

[119] Vasquez ÁR (2020). Pursuit of Harm Reduction in the Alaskan Context: Patient Cultural Explanatory Models of Addiction and Treatment Outcomes for a Medically-Assisted Program Utilizing a Buprenorphine/Naloxone Formulation - ProQuest.

[120] Hewell VM, Vasquez AR, Rivkin ID (2017). Systemic and individual factors in the buprenorphine treatment-seeking process: a qualitative study. Subst Abuse Treat Prev Policy.

[121] Campbell K, McKnight L, Vasquez AR (2018). Integrated primary care‐behavioral health program development and implementation in a rural context. Family Med Community Health.

[122] Freeman LK, Haney AM, Griffin SA (2023). Agreement between momentary and retrospective reports of cannabis use and alcohol use: comparison of ecological momentary assessment and timeline followback indices. Psychol Addict Behav.

