# Supplementary material for: Telehealth-Delivered Mindfulness-Based Intervention: Protocol for a Randomized Clinical Trial for Individuals With Alcohol Use Disorder
Source: JMIR Res Protoc. 2026 May 1;15:e92198. doi: 10.2196/92198 (PMC13134644; doi:10.2196/92198)
Supplement: Peer Review Report 1 [file resprot-v15-e92198-s001.pdf]

1R01AA031159-01 WITKIEWITZ, KATIE

RESUME AND SUMMARY OF DISCUSSION: This new R01 application will evaluate the effectiveness of mindfulness-based relapse prevention (MBRP) via video conferencing as a care option to support long-term recovery from alcohol use disorder (AUD). If successful, the knowledge gained form these studies may help to reduce the public health burden associated with alcohol misuse. The significance of the work is potentially high as there is the need for low-cost accessible AUD continuing care options that focus on harm reduction outcomes in addition to abstinence. The application is conceptually innovative in proposing the telehealth application o mindfulness-based relapse prevention (MBRP) and testing the NIAAA definition of non-abstinent recovery. The research strategy is well-designed: it includes the fully remote nature allowing participation among typically harder to reach rural residing individuals and also describes the use of an effectiveness-implementation design to identify facilitators and barriers to MBRP engagement, adoption, and implementation, and its availability in Spanish and English. The principal investigator has a track record of success working in the field, and the investigative team has been very productive over the recent years. The research environments where the work will be conducted were viewed as highly supportive to the success of the project. However, some weaknesses were identified. Reviewers noted that the effect sizes for superiority of MBRP versus regular treatment are modest in magnitude. In that regard, the findings in a cited bibliography (Grant et al., 2017) did not show significant differences on most outcomes. Since some participants in the mutual support condition will be recruited from ongoing mutual support groups (InTheRooms, SMART recovery), it is unclear whether participants in the MBRP condition would also be recruited from ongoing MBRP sessions and whether these would be matched on length of recovery prior to joining the study. There is lack of details in the approach, which makes difficult to determine if the experimental design and methods are planned with rigor to ensure robust and unbiased results. Following the discussion, the review panel rated the application in the excellent range.

DESCRIPTION (provided by applicant): Alcohol use disorder (AUD) is prevalent and costly, and associated with significant morbidity and mortality. Effective pharmacological and psychosocial treatments for AUD exist, although many individuals do not receive medications, and most are treated via mutual support group participation. Alcoholics Anonymous and other mutual support programs have been shown to be highly effective in supporting abstinence, and they are a tremendously valuable option for those interested in abstinence-based recovery. Yet approximately 80% of individuals with AUD never seek treatment and not wanting to stop drinking is a common barrier to seeking treatment. AA and other mutual support programs are often abstinence-based, yet programs that focus on reductions in drinking have been shown to be as effective at reducing harms related to alcohol use. Recent studies in population-based and clinical samples indicate significant health benefits from drinking reductions, without total abstinence. Aligned with these findings, the National Institute on Alcohol Abuse and Alcoholism has proposed a new operational definition of recovery defined as remission from AUD, cessation of heavy drinking, and improvements in functioning and well-being. Expanding the definition of recovery to include non-abstinent outcomes could increase treatment seeking among those with AUD who are not willing to abstain but are willing to reduce drinking, and reduce the public health burden of untreated AUD. One program that has considerable promise for promoting AUD recovery is mindfulness-based relapse prevention (MBRP). The efficacy of MBRP for reducing heavy drinking has been shown in several trials. Further, MBRP explicitly targets neurobiologically informed domains of addiction: inhibitory control over behavioral responses (executive function); craving and cue reactivity (incentive salience); and negative affect (negative emotionality). MBRP may also be more effective than existing continuing care options in targeting broader health and life functioning. MBRP also has the potential to be broadly accessible via video conferencing. The goal of this study is to examine the effectiveness of MBRP groups delivered via video conferencing in promoting whole-person recovery from AUD up to three years following an attempt to change or stop drinking via treatment or self-change, as compared to referral to online mutual support groups. This study will also examine how MBRP affects mechanisms of behavior change based on

neurobiologically-informed addiction cycle domains. We will use an effectiveness-implementation design to prospectively test the effectiveness of MBRP, as well as identify barriers and facilitators of MBRP group participation to inform future implementation of MBRP continuing care. The ultimate goal of this work is to evaluate MBRP via video conferencing as a continuing care option that supports whole-person recovery and targets addiction cycle domains in supporting long-term recovery from AUD in communities nationwide, including a focus on underserved areas. Availability of effective, evidence-based continuing care interventions may reduce the public health burden of AUD.

PUBLIC HEALTH RELEVANCE: To address the public health burden of alcohol use disorder (AUD), there is a tremendous need for continuing care options to support long-term recovery from AUD that (1) support whole-person recovery, defined as focusing on both functioning and reductions in drinking or abstinence, (2) are effective in targeting the neuroadaptations observed among those with addiction, and (3) are accessible in all communities nationwide, including medically underserved and health professional shortage areas. This project will evaluate the effectiveness and mechanisms of mindfulness-based relapse prevention (MBRP) delivered via video conferencing, as compared to referral to online mutual support groups, in supporting long-term whole-person recovery and improvements in neurobiologically-informed domains of addiction among individuals with AUD who have recently made a change attempt to reduce or stop drinking. The project will also examine the reach, effectiveness, adoption, implementation, and maintenance of MBRP as an accessible and freely available continuing care option that supports long-term recovery from AUD in all communities nationwide, including medically underserved and health professional shortage area

CRITIQUE 1

Significance: 2
Investigator(s): 1
Innovation: 2
Approach: 4
Environment: 1

Overall Impact:

Mindfulness Based Relapse Prevention has shown promise as an aftercare intervention for individuals with Alcohol Use Disorder (AUD). The proposed study will compare it to mutual support groups, with both conditions offered virtually and in an open group format. If MBRP delivered in this manner is demonstrated to facilitate recovery (based on the new operational definition from NIAAA), it would add significantly to available and easily accessible continuing care options. The study has a number of innovative aspects, among which are the inclusion both of individuals who have been in treatment and of individuals who reduced their drinking on their own. It is also innovative in using a national recruitment method. The team of investigators is very strong, and the environment is highly supportive. Enthusiasm is mitigated by several factors, including concern that the inclusion criteria are not completely clear. In addition, there are concerns with recruiting from mutual support options which are also included in the control condition to which they may be randomized.

1. Significance:

Strengths

- There is a significant need for more effective aftercare options for individuals with AUD, given the high rates of relapse. In addition, the requirement for an abstinence goal in most existing mutual support aftercare options likely limits participation by individuals with a drinking reduction goal. Therefore, establishing the efficacy of a non-abstinence-based aftercare option, such as Mindfulness-Based Relapse Prevention, would significantly broaden the network of available supportive aftercare options.

- There is some evidence to support the efficacy of MBRP compared to treatment-as-usual in a few randomized clinical trials.
- Comparing MBRP to existing mutual support groups is reasonable, given their broad accessibility.
- Including participants who reduce drinking without formal treatment is important because they represent the majority of those who quit or reduce alcohol use.
- The addiction cycle constructs that will be examined as mechanisms of behavior change in the proposed study, have been shown in the PI's earlier work to be associated with outcomes in secondary analyses of other studies. This study would test them prospectively.

Weaknesses

- Effect sizes for the superiority of MBRP versus treatment-as-usual are modest in magnitude, and one review (Grant et al., 2017) found no significant differences in most outcome measures.

2. Investigator(s):

Strengths

- The team of investigators and consultants is very strong. Many have extensive publication histories. PI, Dr. Witkiewitz is an expert in AUD, clinical trials, and quantitative methods such as those to be used in this study. She has led previous studies of MBRP.
- Co-Investigators Kirouac, Pearson, Schwebel, and Vasquez bring expertise in rolling admission MBRP groups, qualitative and quantitative methods, mechanisms of behavior change, online support groups, and adapting treatment for Spanish-speaking participants.
- Consultant Bowen is the lead author of the MBRP therapist manual and has trained multiple providers. Drs. Roos and Vinci add experience in the areas of rolling admissions, and remote recruitment and intervention.
- Team members have a history of collaboration.

Weaknesses

- It is not clear who will conduct the qualitative interviews.

3. Innovation:

Strengths

- Numerous aspects of this study are innovative. These include: 1) the combination of the newer (broader) definition of recovery studied longitudinally in both treated and self-changing individuals; 2) long-term study of open, rolling admissions, group MBRP; 3) nationwide recruitment; 4) evaluating implementation facilitators and barriers for remotely delivered, rolling group MBRP for AUD.

Weaknesses

- Some of the elements noted as innovative strengths have been studied in a more piecemeal fashion, in which case the innovation lies in their integrated study.

4. Approach:

Strengths

- The randomized controlled trial of remotely delivered (Zoom) MBRP groups versus online support will provide an ecologically valid test of this type of MBRP as a continuing care intervention applicable to individuals with abstinence or non-abstinence goals.

- Including repeated measures of the proposed mechanisms of change, themselves based on constructs that are key to the addiction cycle, will enable the investigators to determine whether changes in those measures lead to subsequent recovery.
- Self-report of recent alcohol use will be biochemically confirmed at baseline and at the final assessment using PEth via dried blood cards.
- Including social determinants of health will provide contextual variables that are important to take into account when evaluating interventions.
- Sample size should provide adequate power for statistical analyses.

Weaknesses

- There is some ambiguity in the inclusion criteria. In C3.1, the investigators first say that participants will be individuals who have recently stopped or reduced drinking via treatment or self-change. However, in the next paragraph and also in the Protocol Synopsis, the participants are described as people who have either received AUD treatment or reduced or stopped drinking without treatment. The question is whether those who have entered treatment must also have reported a cessation or reduction of drinking. If not, it is difficult to see how the MBRP being tested constitutes “continuing care.
- Exclusion criteria listed in the protocol synopsis (p. 90) include psychosis, mania, and a substance use disorder requiring a higher level of care. How will these be assessed at baseline? What about suicidality?
- Of the three potential online mutual support programs listed in section C3.3, from which those randomized to this condition can choose, LifeRing is abstinence-based, and the rooms have mostly 12-step options, although some other groups are also accessible there. SMART Recovery seems more flexible about the abstinence goal. But if individuals are recruited from SMART Recovery, unless they simply return to that program, their options for non-abstinence-based groups will be limited. Given the flexibility of MBRP regarding abstinence or non-abstinence goals, it seems the options for control participants should assure similar flexibility. Otherwise, different intervention outcomes between MBRP and online support could be attributed to different available goal options.
- Recruitment from potential control condition programs is somewhat problematic. There is some risk that individuals recruited from those sources will not want to re-enter them if they volunteer for this study due to dissatisfaction with their current support group.
- Will facilitators recruited from the community be asked to commit to the study for a minimum length of time? Although they will receive valuable training, supervision and CE opportunities, the lack of financial compensation for facilitating MBRP sessions may make it challenging for them to continue for a lengthy period.
- Fifteen group facilitators will participate in the qualitative interviews. It is not clear how many study participants will be interviewed.
- Not clear how/when alcohol saliva strips will be used.

5. Environment:

Strengths

- The Center on Alcohol, Substance use, and Addictions at the University of New Mexico, and the Department of Psychology, will provide an intellectually and practically supportive environment for this study. The investigators will have the required space, computer resources, and grant management resources for the study.

- Other personnel who will handle evaluation, research coordination, recruitment, quality assurance, and administrative support are in place.

Weaknesses

- None noted.

Study Timeline:

Strengths

- The study timeline has assessments ending in the middle of year 5, allowing 6 months for final data analyses.

Weaknesses

- Recruitment of 430 participants in 18 months is ambitious (6 per week).

Protections for Human Subjects

Acceptable Risks and/or Adequate Protections

- Adequate protection against risks for participants.

Data and Safety Monitoring Plan (Applicable for Clinical Trials Only):

Acceptable

- Includes a DSMB.

Inclusion Plans

- Sex/Gender: Distribution justified scientifically.
- Race/Ethnicity: Distribution justified scientifically.
- Inclusion/Exclusion Based on Age: Distribution justified scientifically.
- Acceptable representation of women, minorities, and adults of all ages over 18.

Resource Sharing Plans:

Acceptable

Budget and Period of Support

Recommend as Requested

CRITIQUE 2

Significance: 2
Investigator(s): 2
Innovation: 2
Approach: 3
Environment: 1

Overall Impact:

This R01 application proposes a hybrid implementation-effectiveness design to examine the feasibility and effectiveness of mindfulness-based relapse prevention (MBRP) groups delivered via video conferencing in promoting whole-person recovery from AUD in persons up to three years following an attempt to change or stop drinking via treatment or self-change. N = 430 persons with recent attempts at change in drinking behavior will be randomized to MBRP vs. referral to online mutual support groups. Measures of alcohol and other substance use as well as psychosocial functioning, predictors of recovery, and addiction cycle domains (executive functioning, self-efficacy, incentive salience, and negative emotions) will be assessed every 6 months x 3 years. MBPR is hypothesized to be superior to mutual support in all domains. Additionally, the feasibility of virtually delivered MBRP will be assessed via mixed methods. The research question is significant, and the investigators clearly possess the expertise to complete the project. There are some minor concerns related to innovation and the approach.

1. Significance:

Strengths

- Alcohol use disorder (AUD) is prevalent and costly and associated with significant morbidity and mortality.
- Effective pharmacological and psychosocial treatments for AUD exist but only a minority of people access these treatments.
- Recent studies in population-based and clinical samples indicate significant health benefit from drinking reductions, without total abstinence.
- There is a need for more continuing care options to support sustained recovery, including for those who are not interested in complete abstinence.
- Continuing care options should broaden the focus beyond abstinence from alcohol to improvement in functioning.
- Mindfulness-based relapse prevention (MBRP) has shown promise for the reduction of drinking in AUD.

Weaknesses

- MBRP's efficacy in the reduction of heavy drinking has previously been demonstrated in RCTs.

2. Investigator(s):

Strengths

- Track record of prior collaboration between investigators.
- Principal investigator is a Distinguished Professor of Psychology and Director of the Center on Alcohol, Substance Use and Addictions at the University of New Mexico and has an extensive quantitative methodology and alcohol-related intervention experience, including trials of MBRP.
- Co-I Kirouac has training in mindfulness-based treatments for SUD and expertise in non-abstinence-based recovery and assessment of psychosocial functioning as part of broader AUD recovery definitions.
- Co-I Pearson has expertise in mechanisms of behavior change and technology-delivered behavioral interventions, with emphasis on the use of ecological momentary assessment for data collection. Has additional expertise in advanced quantitative methods.
- Co-Schwebel is a post-doctoral fellow focusing on mHealth interventions for SUD. Of note, he is a bilingual licensed clinical psychologist and will assist with the delivery of the MBPR in Spanish, thereby increasing access to an ethnically minoritized community.

- Co-I Vasquez is a clinical-community psychologist with expertise in addressing addiction recovery in diverse populations, CBPR, and cultural adaption of existing behavioral interventions.
- Consultant Bowen has extensive experience with MBRP in different populations and in training other professionals to deliver MBRP.
- Consultant Roos is an Early-Stage Investigator and has prior experience with MBRP delivery, primarily focusing on increasing scalability and access, including MBPR delivered via technology- telehealth and apps.
- Consultant Vinci has expertise in behavioral interventions, with an emphasis on mHealth to develop and test interventions for substance use disorders. Recently completed R34 using MBRP via telehealth.

Weaknesses

- There is no a biostatistician in the supportive team.

3. Innovation:

Strengths

- Testing updated definition of non-abstinent recovery in longitudinal sample.
- MBPR delivered via telehealth.
- Comparison of MBRP as a continuing care intervention vs. mutual support in no abstinent recovery.

Weaknesses

- Team has already piloted Zoom delivery of MBRP and is using it in 2 multi-site trials.

4. Approach:

Strengths

- Long-term follow-up (3 years).
- Nationwide recruitment of a diverse study population with the delivery of the intervention in English and Spanish.
- Assessment of both individual and community-level determinants of recovery.
- Inclusion of a biomarker.

Weaknesses

- While groups will be balanced on AUD severity, they will not be balanced on heavy drinking patterns. AUD severity and drink quantity/frequency are not necessarily equivalent.
- No description of orienting those referred to mutual support on alcohol saliva strips or blood spot data collection.
- No description of how mutual support participation would be assessed.
- Individual’s insurance status was not included in the demographics data or in the economic resources list as a measure of SDOH.
- Data collection does not appear to capture or control for use of pharmacotherapy for AUD.

- While a biomarker is used to confirm self-reported alcohol use, it is only administered at baseline and at the end of the 3-year study. Would anticipate more utility of the results if administered more frequently, at least annually, throughout the study.
- Unclear how a dose of the intervention and control group will be measured and included in the analyses (# of sessions attended or frequency of attendance).

5. Environment:

Strengths

- Site includes a university-wide SUD research center with 30-year history.

Weaknesses

- No concerns

Study Timeline:

Strengths

- Already have several facilitators trained in delivering the study intervention.

Weaknesses

- None noted.

Protections for Human Subjects

Acceptable Risks and/or Adequate Protections

Data and Safety Monitoring Plan (Applicable for Clinical Trials Only):

Acceptable

- o Does include a plan for a DSMB. While data will be submitted to the DSMB quarterly, there is no clarity on how often the DSMB members will actually meet.

Inclusion Plans

- Sex/Gender: Distribution justified scientifically.
- Race/Ethnicity: Distribution justified scientifically.
- Inclusion/Exclusion Based on Age: Distribution justified scientifically.
- Acceptable representation of women, minorities, and adults of all ages over 18.
- Includes young adults 18-20.

Resource Sharing Plans:

Acceptable

Budget and Period of Support

Recommend as Requested

CRITIQUE 3

Significance: 2
Investigator(s): 1
Innovation: 1
Approach: 3
Environment: 1

Overall Impact:

This new R01 proposes to test the effectiveness of mindfulness-based relapse prevention (MBRP) delivered by videoconferencing to promote long-term AUD recovery (over 3 years post change in drinking) in comparison to referral to online mutual support groups. The research will also assess the mechanisms of behavior change produced by MBRP by assessing three distinct domains relevant to addiction processes: inhibitory control/executive function, craving/incentive salience, and negative affect/negative emotionality. In addition, community-level variables will be assessed. Finally, the research will use an effectiveness-implementation design to examine facilitators and barriers to participating in MBR, and also the uptake, implementation, and effectiveness of MBRP for continuing care throughout the US. Expected outcomes are that AUD remission, cessation of heavy drinking (confirmed by blood PEth), and improvements in functioning and well-being will be retained at 3 years among groups of individuals who received MBRP in comparison to referral to online mutual support groups. This is a novel and timely research proposal from a very strong and experienced investigative group at the University of New Mexico. The significance lies in the necessity for low cost widely available continuing care programs for AUD recovery that focus on harm reduction outcomes instead of abstinence. The rationale is strong and based on empirical evidence demonstrating the effectiveness of MBRP to reduce heavy drinking and also theoretical models of processes important in AUD. The trial is well-designed and innovative in many aspects including the fully remote nature allowing participation in a wide range of areas (metropolitan, urban, suburban, rural, remote) across the US, and the use of an effectiveness-implementation design to identify facilitators and barriers to access. This is a very strong proposal likely to have a significant impact on the field and on public health. I have only minor comments regarding procedural aspects.

1. Significance:

Strengths

- MBRP may represent a new low cost widely available continuing care effort for AUD recovery that does not focus on maintenance of abstinence.
- MBRP will promote whole-person recovery and life functioning by targeting distinct pathological constructs; increases inhibitory control and reduces automaticity by promoting awareness, reduces craving using an approach called urge surfing, reduces overall distress by encouraging individuals to experience the time-limiting nature of unpleasant emotional states.
- Mechanisms of action of MBRP are expected to be mediated by separate domains of addiction processes including improved executive function (better inhibitory control of behavior), diminished incentive salience (craving), and less negative emotionality (negative affect).
- Scientific rationale based on evidence from RCTs that demonstrate the effectiveness of MBRP to maintain reduced heavy drinking with the largest effects observed when MBRP is delivered after treatment. Feasibility of remote delivery demonstrated in a pilot trial.
- MBRP group facilitators offer services free of charge in line with Buddhist principles from where mindfulness meditation originates.
- The study will be conducted remotely nationwide using an effectiveness-implementation hybrid design to speed the uptake of MBRP into practice.

Weaknesses

- Is it not known whether uptake and implementation of MBRP by other practitioners will offer services free of charge (esp. for-profit private practitioners, treatment programs)?

2. Investigator(s):

Strengths

- Dr. Katie Witkiewitz PD/PI is a Distinguished Professor of Psychology and Director of the Center on Alcohol, Substance use, And Addictions (CASAA) at the University of New Mexico. She has expertise in the treatment of AUD/SUD, promoting harm reduction goals for AUD recovery, and mechanisms of behavior change. She pioneered the use of mindfulness-based treatments for AUD/SUD, developed MBRP for treatment recovery, and has worked to develop MBRP to be delivered virtually via videoconferencing. She has the necessary experience and is ideally placed to successfully oversee this plan of research.
- Dr. Megan Kirouac (Co-Investigator) is a clinical psychologist and Research Assistant Professor in the CASAA at the University of New Mexico. She has experience conducting MBRP sessions both in-person and remotely. Her role on the project is to assist the PI with all aspects of the research, supervise MBRP group facilitators, assess the fidelity of the MBRP program, respond to any clinical emergencies, and contribute to presentations and manuscripts.
- Dr. Matthew Pearson (Co-Investigator) is a Research Professor in the Department of Psychology at the University of New Mexico. He has specific expertise in studying mechanisms of behavior change in SUD/AUD using remote technology (EMA) and experience in advanced statistical modeling. His role includes randomization of participants, overseeing data collection, assisting with data analysis, and preparing presentations and manuscripts.
- Dr. Frank Schwebel (Co-Investigator) is a Research Assistant Professor in the Department of Psychology at the University of New Mexico. He has prior experience conducting MBRP for SUD including in-person and also remote sessions and also developed a Spanish-language version of the treatment. He will be responsible for supervising MRBP sessions conducted in Spanish and ensuring fidelity.
- Dr. Angel Vasquez (Co-Investigator) is a clinical-community psychologist and Clinical Research Associate at CASAA at the University of New Mexico with specific expertise in engaging and working with minority populations and historically under-served communities to address addiction recovery and comorbid health conditions. For the project, he will focus on community engagement, qualitative methods, and tailoring MBRP for Spanish speakers and other cultural factors.
- The team will be assisted by expert consultants with expertise in MBRP and who has previously collaborated with the PI. Sarah Bowen (Consultant) is an Associate Professor of Psychology at Pacific University with specific expertise in MBRP, having developed and authored the MBRP therapist manual and helped with conducting the first RCTs of this approach. Corey Roos (Consultant) is an Assistant Professor at Yale University with expertise in developing mindfulness treatment approaches for disorders including SUD. Christine Vinci (Consultant) is an Assistant Member of the Department of Health Outcomes and Behavior at the Moffitt Cancer Center. She has specific expertise in mhealth technologies and the delivery of mindfulness-based treatments remotely to reduce substance use.
- There is an established record of collaborations between the investigators on the team and each has a clearly defined role in the research.

Weaknesses

- None noted.

3. Innovation:

Strengths

- Applying MBRP to long-term AUD recovery care delivered remotely via videoconferencing is novel.
- MBRP will be one of the first continuing care approaches that do not promote abstinence goals and promote overall well-being and life functioning.
- Targeting multiple distinct pathophysiological processes in AUD is novel.
- Completely remote procedures including recruitment, consent, and data collection are innovative and enable reach to diverse areas including rural communities and metropolitan areas.

Weaknesses

- None noted.

4. Approach:

Strengths

- Recruitment of equal numbers of men and women and over recruitment of individuals from minority populations and Spanish speakers.
- The effectiveness-implementation hybrid design will allow the investigators to assess facilitators and barriers to individuals participating in the program.
- Groups will be matched on sex, the severity of AUD, initial recovery goal (abstinence vs. reduction), and age using urn randomization.
- Participants can join as many MBRP sessions as they like for as long as they like.
- Biological analyses of saliva and blood to confirm self-reported alcohol consumption.
- Comprehensive assessment of self-report measures (alcohol use problems and craving, AUD score, life functioning, incentive salience, executive function, negative emotionality, treatment history, continuing care ratings) and biological samples (blood spots for PEth analysis) are collected at baseline and every 6 months.

Weaknesses

- Is the total sample number 430 (as stated in Specific Aims) or 860 i.e., 430 per group, as stated in the 'Research Strategy; Sample size considerations, statistical power, and retention section'?.
- Are the MBRP and mutual support group sessions an equivalent length? Will attendance at sessions be tracked in either condition and verified? There is the potential for group differences in exposure to virtual group settings. That is, participants in the mutual support group condition are referred but may not attend.
- Are facilitators located and also trained remotely? How will the personal mindfulness practice of facilitators be confirmed?
- Some participants in the mutual support groups condition will be recruited from ongoing mutual support groups (InTheRooms, SMART recovery). Will participants in the other condition also be recruited from ongoing MBRP sessions? Will these participants be matched on length of recovery prior to joining the study, AUD severity, consumption before change and goals of treatment?
- Different people may have the same score for AUD but differ in drinking patterns, for example daily heavy drinking vs. light or no drinking for several days followed by a very heavy binge. Is

there a way to balance patterns between conditions or to examine recovery among drinker subtypes who exhibit different drinking patterns before change?

- There may be bias in the qualitative interviews of participants as those who discontinue the MBRP sessions without notifying facilitators will be lost to follow-up. Could interviews be conducted at a defined time after starting the program, say 12 or 18mo to try to mitigate any bias? Conducting interviews with facilitators and participants sooner than at the end of the study will also identify any significant barriers earlier.

5. Environment:

Strengths

- Center on Alcohol, Substance use, And Addictions (CASAA) has the necessary facilities and equipment for the research and UNM is a supportive environment.

Weaknesses

- None noted.

Study Timeline:

Strengths

- Appears feasible given the expertise of the team and that they are already familiar with the procedures.

Weaknesses

- None noted

Protections for Human Subjects

Acceptable Risks and/or Adequate Protections

Data and Safety Monitoring Plan (Applicable for Clinical Trials Only):

  - Acceptable.

Inclusion Plans

- Sex/Gender: Distribution justified scientifically.
- Race/Ethnicity: Distribution justified scientifically.
- Inclusion/Exclusion Based on Age: Distribution justified scientifically.

Resource Sharing Plans:

Acceptable

Budget and Period of Support

Recommend as Requested

THE FOLLOWING SECTIONS WERE PREPARED BY THE SCIENTIFIC REVIEW OFFICER TO SUMMARIZE THE OUTCOME OF DISCUSSIONS OF THE REVIEW COMMITTEE, OR REVIEWERS' WRITTEN CRITIQUES, ON THE FOLLOWING ISSUES:

PROTECTION OF HUMAN SUBJECTS: ACCEPTABLE

INCLUSION OF WOMEN PLAN: ACCEPTABLE

INCLUSION OF MINORITIES PLAN: ACCEPTABLE

INCLUSION ACROSS THE LIFESPAN: ACCEPTABLE

COMMITTEE BUDGET RECOMMENDATIONS: The budget was recommended as requested.

Footnotes for 1 R01 AA031159-01; PI Name: Witkiewitz, Katie A

Ad hoc or special section application percentiled against "Total CSR" base.

NIH has modified its policy regarding the receipt of resubmissions (amended applications).See Guide Notice NOT-OD-18-197 at https://grants.nih.gov/grants/guide/notice-files/NOT-OD-18-197.html. The impact/priority score is calculated after discussion of an application by averaging the overall scores (1-9) given by all voting reviewers on the committee and multiplying by 10. The criterion scores are submitted prior to the meeting by the individual reviewers assigned to an application, and are not discussed specifically at the review meeting or calculated into the overall impact score. Some applications also receive a percentile ranking. For details on the review process, see http://grants.nih.gov/grants/peer_review_process.htm#scoring.

MEETING ROSTER

Clinical, Treatment and Health Services Research Study Section
National Institute on Alcohol Abuse and Alcoholism Initial Review Group
NATIONAL INSTITUTE ON ALCOHOL ABUSE AND ALCOHOLISM
AA-3
02/08/2023

Notice of NIH Policy to All Applicants: Meeting rosters are provided for information purposes only. Applicant investigators and institutional officials must not communicate directly with study section members about an application before or after the review. Failure to observe this policy will create a serious breach of integrity in the peer review process, and may lead to actions outlined in NOT-OD-22-044 at https://grants.nih.gov/grants/guide/notice-files/NOT-OD-22-044.html, including removal of the application from immediate review.

CHAIRPERSON(S)

KELLY, JOHN F., PHD
ELIZABETH R. SPALLIN PROFESSOR OF PSYCHIATRY
HARVARD MEDICAL SCHOOL
DIRECTOR, MGH RECOVERY RESEARCH INSTITUTE
ASSOCIATE DIRECTOR, MGH CENTER FOR ADDICTION
BOSTON, MA 02114

MEMBERS

ALVANZO, ANIKA A.H., MD
EASTERN REGION MEDICAL DIRECTOR
PYRAMID HEALTHCARE, INC.
DUNCANVILLE, PA 16635

ARIAS, ALBERT JOSEPH, MD
ASSOCIATE PROFESSOR
DEPARTMENT OF PSYCHIATRY
DIVISION OF ADDICTION PSYCHIATRY
VIRGINIA COMMONWEALTH UNIVERSITY
RICHMOND, VA 23284

CHILDS, EMMA, PHD
ASSOCIATE PROFESSOR
DEPARTMENT OF PSYCHIATRY
THE UNIVERSITY OF ILLINOIS AT CHICAGO
CHICAGO, IL 60612

CURRY, JOHN F, PHD
PROFESSOR EMERITUS
DEPARTMENT OF PSYCHIATRY & BEHAVIORAL SCIENCES
DUKE UNIVERSITY
DURHAM, NC 27705-5696

DEVINE, ERIC G., PHD
ASSISTANT PROFESSOR OF PSYCHIATRY AND DIRECTOR
CLINICAL STUDIES UNIT
DEPARTMENT OF PSYCHIATRY
BOSTON UNIVERSITY CHOBANIAN & AVEDISIAN
SCHOOL OF MEDICINE
BOSTON, MA 02118

GILBERT, PAUL A., PHD
ASSOCIATE PROFESSOR
DEPARTMENT OF COMMUNITY AND BEHAVIORAL HEALTH
UNIVERSITY OF IOWA COLLEGE OF PUBLIC HEALTH
IOWA CITY, IA 52242

JORDAN, AYANA, MD, PHD
ASSOCIATE PROFESSOR OF PSYCHIATRY
DEPARTMENT OF PSYCHIATRY
NYU GROSSMAN SCHOOL OF MEDICINE
NEW YORK, NY 10016

LINDGREN, KRISTEN P., PHD
PROFESSOR
DEPARTMENT OF PSYCHIATRY AND BEHAVIORAL SCIENCE
UNIVERSITY OF WASHINGTON
SEATTLE, WA 98195-6560

MERICLE, AMY A., PHD
RESEARCH SCIENTIST
ALCOHOL RESEARCH GROUP
PUBLIC HEALTH INSTITUTE
EMERYVILLE, CA 94608

SCHACHT, JOSEPH P., PHD
ASSOCIATE PROFESSOR
DEPARTMENT OF PSYCHIATRY
UNIVERSITY OF COLORADO SCHOOL OF MEDICINE
AURORA, CO 80045

STERLING, STACY ANN, DRPH
CO-DIRECTOR, CENTER FOR ADDICTION AND MENTAL
HEALTH RESEARCH ASSOCIATE PROFESSOR, DEPT OF
HEALTH SYSTEMS SCIENCE
DEPARTMENT OF HEALTH SYSTEMS SCIENCE
KAISER PERMANENTE
BERNARD J. TYSON SCHOOL OF MEDICINE
OAKLAND, CA 94612

SCIENTIFIC REVIEW OFFICER

ESPINOZA, LUIS, PHD
SCIENTIFIC REVIEW OFFICER
EXTRAMURAL PROJECT REVIEW BRANCH
NATIONAL INSTITUTE ON ALCOHOL ABUSE AND ALCOHOLISM
NATIONAL INSTITUTES OF HEALTH
BETHESDA, MD 20817

EXTRAMURAL SUPPORT ASSISTANT

LYLES, DEIRDRE
EXTRAMURAL SUPPORT ASSISTANCE
OFFICE OF EXTRAMURAL ACTIVITIES, EPRB
NATIONAL INSTITUTE OF ALCOHOL ABUSE AND ALCOHOLISM
NATIONAL INSTITUTES OF HEALTH
BETHESDA, MD 20892-6902

Consultants are required to absent themselves from the room during the review of any application if their presence would constitute or appear to constitute a conflict of interest.
